# Cooperative treatment effectiveness of ATR and HSP90 inhibition in Ewing’s sarcoma cells

**DOI:** 10.1186/s13578-021-00571-y

**Published:** 2021-03-20

**Authors:** Christian Marx, Marc U. Schaarschmidt, Joanna Kirkpatrick, Lisa Marx-Blümel, Melisa Halilovic, Martin Westermann, Doerte Hoelzer, Felix B. Meyer, Yibo Geng, Katrin Buder, Hauke M. Schadwinkel, Kanstantsin Siniuk, Sabine Becker, René Thierbach, James F. Beck, Jürgen Sonnemann, Zhao-Qi Wang

**Affiliations:** 1 Leibniz Institute On Aging - Fritz Lipmann Institute (FLI), Jena, Germany; 2 Department of Pediatric Hematology and Oncology, Children’s Clinic, Jena University Hospital, Jena, Germany; 3 Research Center Lobeda, Jena University Hospital, Jena, Germany; 4 Electron Microscopy Center, Jena University Hospital, Jena, Germany; 5 Department of Human Nutrition, Institute of Nutrition, Friedrich Schiller University Jena, Jena, Germany; 6 Department of Neurosurgery, Beijing Tiantan Hospital, Capital Medical University, Beijing, China; 7 Faculty of Biology and Pharmacy, Friedrich Schiller University of Jena, Jena, Germany; 8 Present Address: Francis Crick Institute, London, UK; 9 Present Address: Biopharmaceutical New Technologies (BioNTech) Corporation, Mainz, Germany; 10 Klinik Für Kinder- Und Jugendmedizin, Universitätsklinikum Jena, Am Klinikum 1, 07747 Jena, Germany

**Keywords:** ATM, ATR, Ewing's sarcoma, HSP90, Endoplasmic reticulum (ER) stress, Apoptosis

## Abstract

**Introduction:**

Ewing's sarcoma is an aggressive childhood malignancy whose outcome has not substantially improved over the last two decades. In this study, combination treatments of the HSP90 inhibitor AUY922 with either the ATR inhibitor VE821 or the ATM inhibitor KU55933 were investigated for their effectiveness in Ewing's sarcoma cells.

**Methods:**

Effects were determined in p53 wild-type and p53 null Ewing's sarcoma cell lines by flow cytometric analyses of cell death, mitochondrial depolarization and cell-cycle distribution as well as fluorescence and transmission electron microscopy. They were molecularly characterized by gene and protein expression profiling, and by quantitative whole proteome analysis.

**Results:**

AUY922 alone induced DNA damage, apoptosis and ER stress, while reducing the abundance of DNA repair proteins. The combination of AUY922 with VE821 led to strong apoptosis induction independent of the cellular p53 status, yet based on different molecular mechanisms. p53 wild-type cells activated pro-apoptotic gene transcription and underwent mitochondria-mediated apoptosis, while p53 null cells accumulated higher levels of DNA damage, ER stress and autophagy, eventually leading to apoptosis. Impaired PI3K/AKT/mTOR signaling further contributed to the antineoplastic combination effects of AUY922 and VE821. In contrast, the combination of AUY922 with KU55933 did not produce a cooperative effect.

**Conclusion:**

Our study reveals that HSP90 and ATR inhibitor combination treatment may be an effective therapeutic approach for Ewing's sarcoma irrespective of the p53 status.

**Supplementary Information:**

The online version contains supplementary material available at 10.1186/s13578-021-00571-y.

## Introduction

Ewing's sarcoma (ES) is the second most frequent bone cancer during childhood and adolescence [1, 2]. 85% of ES cases arise from a translocation of the *EWSR1* gene with the *FLI1* gene, resulting in the fusion oncogene *EWS-FLI1*. While EWSR1 participates in the DNA damage response (DDR) by recruiting BRCA1 to resolve DNA:RNA hybrid structures, so-called R-loops, and for homologous recombination (HR) [1, 3, 4], EWS-FLI1 prevents BRCA1-dependent DNA repair. Therefore, ES, although not harboring BRCA1 or BRCA2 mutations, behaves like BRCA-deficient tumors, rendering ES exceptionally vulnerable to DNA-damaging treatments [4]. Additional mutations in ES are rare, with *STAG2*, *CDKN2A* and *TP53* being the most frequently mutated genes [1]. Yet, it should be noted that the ~ 10% of ES patients with mutant p53 have a particularly poor outcome [5, 6].

The therapy for ES consists of neoadjuvant chemotherapy, surgery and/or radiation of the tumor area, followed by a multimodal treatment regimen with vincristine, doxorubicin, cyclophosphamide, ifosfamide and/or etoposide [1, 2]. However, approximately 25% of the patients with metastasis at diagnosis are resistant to intensive therapies [1]. The recurrence rates for patients with initially localized tumors are about 25% and for patients with initially metastatic tumors 50–80%; of the patients with relapsed disease less than 20% survive [1, 2]. The anti-ES agents that hold promise to increase treatment effectiveness and to overcome resistance include inhibitors of growth factors, epigenetic modifiers, PARP1 inhibitors, p53 activators and PD-L1-based immune therapies [1, 2]. Drugs that target DDR pathways may also be suitable to treat otherwise therapy-resistant ES [4, 7]. However, clinically effective therapeutic strategies are as yet unavailable. Recently, inhibitors of ATR and ATR-mediated pathways, as well as of HSP90 have been shown to be effective in ES in vitro [8–12].

In the present study, we asked if combinations of HSP90 inhibitors (HSP90i) and ATR inhibitors (ATRi) would exceed the cytotoxicity of the individual compounds in ES cells. We used AUY922 (also known as NVP-AUY922 or luminespib), which is one of the most effective HSP90i, and VE821, a potent and specific ATRi. Both AUY922 and the VE821 homolog VE822 (also known as VX-970, M6620 or berzosertib) are tested in clinical phase I/II trials as single drug treatment or in combination with other chemotherapeutics [13, 14]. We found that VE821 strongly enhanced the effectiveness of AUY922 in both p53 wild-type (wt) WE-68 and p53 null (-/-) A673 ES cells, thus offering a novel strategy to target ES cells irrespective of their p53 status.

## Materials and methods

### Cell culture and treatment

Dr. F. van Valen (Münster, Germany) kindly provided WE-68 cells. A673 cells were purchased from Sigma Aldrich and SaOS-2 cells were purchased from the DSMZ. HCT116 p53^wt^ and p53^−/−^ colon cancer cells were a gift from Dr. B. Vogelstein (Baltimore, MD, USA). WE-68 cells were maintained in RPMI 1640, SaOS-2 cells were maintained in McCoy's 5A medium, A673 and HCT116 cells were maintained in DMEM with 4.5 g/l glucose (all from Thermo Fisher). RPMI 1640 and DMEM were supplemented with 10% FCS and McCOY's 5A was supplemented with 15% FCS; all media were supplemented with 2 mM L-glutamine and 100 U/ml penicillin/streptomycin (all from Thermo Fisher). ES cells were cultivated in collagen-coated (5 μg/cm^2^; Thermo Fisher) tissue culture flasks. Dr. A. Poth (Roßdorf, Germany) kindly provided BALB/c-3T3-A31-1–1 cells from Hatano Research Institute of Japan. BALB/c cells were maintained in DMEM/HAM's F-12 (3.0 g/l glucose; Biochrom) supplemented with 5% FCS and 100 U/ml penicillin/streptomycin. Only sub-confluent cells (about 70% confluence) between the passages 20 to 40 were used for the BALB-CTA. All cells were cultivated in a humidified incubator at 37 °C with 5% CO_2_.

Cells were treated with 0–50 mM AUY922 (Luminespib; S1069), 1–10 µM VE821 (S8007), 5–15 µM KU55933 (S1092), 0.4–5 µg/ml tunicamycin (S7894) (all in DMSO and from Selleck Chemicals) or their combinations for up to 72 h.

### Crystal violet (Gentian violet) cell proliferation assay

Crystal violet staining was performed as previously described in [15].

### BALB/c cell transformation assay (BALB-CTA)

The BALB-CTA was performed as previously described in [16].

### ***Flow cytometric analysis of cell death and loss of mitochondrial transmembrane potential (ΔΨ***_***M***_***)***

Analyses were performed as previously described in [17].

### mRNA expression analysis by real-time RT-PCR

Total RNA was isolated using Peqgold Total RNA Kit including DNase digestion (Peqlab). RNA was transcribed into cDNA using Omniscript (Qiagen). Real-time PCR was conducted using the 7900HT Fast Real-Time PCR system (Thermo Fisher). Expression levels were normalized to β-2-microglobulin. Reactions were done in duplicate using Taqman Gene Expression Assays (*BAK1*: Hs00832876_m1, *BAX*: Hs00180269_m1, *BCL2L11*: Hs00708019_s1, *BCL2L1*: Hs00236329_m1, *BCL2*: Hs00608023_m1, *MCL1*: Hs01050896_m1, *CDKN1A*: Hs00355782_m1, *PMAIP1*: Hs00560402_m1, *BBC3*: Hs00248075_m1, *ATR*: Hs00992123_m1, *ATM*: Hs00175892_m1, *CHEK1*: Hs00967506_m1, *CHEK2*: Hs00200485_m1, *AIFM1*: Hs00377585_m1, *TP53*: Hs01034249_m1, *MDM2*: Hs00242813_m1; *β-2-microglobulin*: Hs00187842_m1) and Universal PCR Master Mix (Thermo Fisher). All procedures were carried out according to the manufacturers' protocols. The relative expression levels were calculated by the 2^(−ΔΔCt)^ method.

### Whole cell lysates and immunoblotting

Preparation of whole cell lysates and immunoblotting was performed as previously described in [18]. Antibodies used: ATR (1:1000; 13934S), phospho-S428 ATR (1:2500; 2853S), phospho-S1981 ATM (1:2500; 4526S), ATF-4 (1:1000; 11815S) cleaved caspase-3 (1:1000; 9661S), PARP1 (1:1000; 9542S), phospho-S345 CHK1 (1:1000; 2341S), CHK1 (1:1000; 2345S), CHK2 (1:1000; 2662S), CHOP (1:1000; 2895S), BIM (1:2500; 2933S), LC3B (1:1000; 2775S), phospho-S15 p53 (1:2500; 9284S), phospho-S9 GSK3β (1:2500; 9323S), phospho-S473 AKT (1:2500; 4060S), AKT (1:1000; 2920S), phospho-T389 p70 S6 kinase (1:2500; 9205S), p70 S6 kinase (1:2500; 9202S) (all from Cell Signaling), GRP78 (BiP; 1:1000; AF4846; R&D systems), ATM (1:2500; NB100-309, Novus Biologicals), p53 (1:5000; sc-965), BRCA1 (1:500; sc-6954), DR5 (1:1000; sc-166624), LAMP1 (1:2500; sc-20011) (all from Santa Cruz), p62 (1:2500; PM045, MBL) and phospho-S139 (γH2AX; 1:2500; 07–164, Merck Millipore). Equal loading of protein was verified by the detection of vinculin (1:5000; sc25336, Santa Cruz), β-actin (1:10,000; A5441) and α-tubulin (1:10,000; T5168) (both from Sigma Aldrich). Images were quantified using ImageJ 1.8 (NIH).

### Flow cytometry of cytosolic reactive oxygen species (ROS)

Before harvesting, cells were incubated with 2 μM 6-carboxy-2′,7′-dichlorodihydrofluorescein diacetate (CM-H_2_DCFDA; Thermo Fisher) at 37 °C for 30 min. Cytosolic ROS level were assessed by determining the formation of fluorescent 6-carboxy-2′,7′-dichlorodihydro-fluorescein (CM-DCF). 10,000 cells per sample were analyzed on a FACS Canto II (BD Bioscience); data were gated to exclude debris.

### Flow cytometric analysis of cell cycle profiles

Analyses were performed as previously described in [15].

### Fluorescence microscopy

Cells were fixed with 4% paraformaldehyde (in PBS; Carl Roth) and incubated in blocking solution (BS: 5% donkey serum, 0.4% Triton-X 100 in PBS). Samples were incubated in primary antibody solutions against GM130 (1:500 in BS; 610,822; BD Biosciences) or GRP94 (1:500 in BS; MA3-016) in PBS and incubated in AlexaFluor 555-conjugated anti-rabbit (1:200 in BS; A21434) or AlexaFluor 488-conjugated anti-mouse (1:200 in BS; A21202) (the latter three Invitrogen) secondary antibody solutions. Afterward, samples were stained with 1 µg/ml DAPI (Sigma Aldrich) and mounted with ProLong Gold Antifade Mountant (Thermo Fisher). Images were examined using a Zeiss AxioImager ApoTome microscope (structured illumination) (Carl Zeiss). Zen 2.0 lite software (Carl Zeiss) was used to analyze the pictures.

### Transmission electron microscopy of ES cells

Cells were fixed in culture dishes with 2.5% glutaraldehyde in Seahorse XF DMEM medium (Agilent Technologies; pH 7.4) for 1 h at RT and washed once in Seahorse XF DMEM medium. Cells were harvested by scraping, pelleted for 5 min at 2000 rpm at RT and washed two times in cacodylate buffer (100 mM, pH 7.4). Cell pellets were post-fixed with 1% osmium tetroxide in cacodylate buffer for 2 h, dehydrated in an ascending ethanol series and stained with 2% uranyl acetate in 50% ethanol. The samples were embedded in Araldite resin (Plano) according to the manufacturer's instruction. Ultrathin sections of 70 nm thickness were cut using an ultramicrotome Ultracut E (Reichert-Jung) and mounted on Formvar carbon-coated 100 mesh grids (Quantifoil). The Ultrathin sections were stained with lead citrate for 10 min [19] and examined in a Zeiss CEM 902 A transmission electron microscope (Carl Zeiss). Images were acquired using a 1 k FastScan CCD camera (camera and acquisition software, TVIPS).

### Proteome analysis of A673 cells

Cell pellets (10 million cells per biological replicate from all conditions) were thawed on ice and resuspended in 0.5 ml of PBS. Subsequently, 0.5 ml of a 2 × lysis buffer was added (final 100 mM HEPES, 50 mM DTT, 4% SDS). Protein concentration was expected to be around 1 mg/ml. Cell lysates were sonicated (Bioruptor Plus, Diagenode) with 10 cycles (1 min ON, 30 s OFF, 20 °C), before heating at 95 °C for 10 min. The sonication was repeated once. Following alkylation (15 mM iodoacetamide, 30 min, RT in the dark), 50 µg protein per sample was precipitated in ice-cold acetone (4 × sample volume, overnight, -20 °C). Protein pellets were obtained by centrifugation (20000* g*, 30 min, 4 °C) and were washed twice with 400 µl ice-cold 80% acetone/water. Pellets were vortexed and centrifuged (10 min after first wash, 2 min after second, at 20000* g*, 4 °C), before resuspension by sonication (as described before) in lysis buffer (100 mM HEPES, 3 M urea, pH 8.0) at a concentration of 1 µg/µl. Digestion with Lys-C (1:100 enzyme/protein; Wako) was carried out for 4 h at 37 °C, followed by 1:2 dilution with water and a secondary digestion with trypsin (1:100 enzyme/protein; Promega) performed overnight at 37 °C. Digested peptides were acidified by the addition of 10% TFA to obtain pH 2 and then desalted using an Oasis® HLB μElution Plate (Waters Corporation). Digested peptides were spiked with the indexed retention time peptide (iRT) kit (Biognosys AG) and separated by the nanoAcquity M-Class Ultra-High Performance Liquid Chromatography system (Waters) fitted with a trapping (nanoAcquity Symmetry C18, 5 µm, 180 µm × 20 mm) and an analytical column (nanoAcquity BEH C18, 1.7 µm, 75 µm × 250 mm). The outlet of the analytical column was coupled directly to a Q-Exactive HF-X (Thermo Fisher Scientific) using the Proxeon nanospray source. Solvent A was water, 0.1% FA and solvent B was acetonitrile, 0.1% FA. Samples were loaded at constant flow of solvent A at 5 μl/min onto the trap for 6 min. Peptides were eluted via the analytical column at 0.3 μl/min and introduced via a Pico-Tip Emitter 360 μm OD × 20 μm ID; 10 μm tip (New Objective). A spray voltage of 2.2 kV was used. During the elution step, the percentage of solvent B increased in a non-linear fashion from 0 to 40% in 120 min. Total run time was 145 min. The capillary temperature was set at 300 °C. The RF lens was set to 40%. MS conditions were: full scan MS spectra with mass range 350–1650 m/z was acquired in profile mode in the Orbitrap with resolution of 120,000 FWHM. The filling time was set at maximum of 60 ms with limitation of 3e^6^ ions. DIA scans were acquired with 40 mass window segments of differing 20 widths across the MS1 mass range. The default charge state was set to 3 + . HCD fragmentation (stepped normalized collision energy; 25.5, 27, 30%) was applied and MS/MS spectra were acquired with a resolution of 30,000 FWHM with a fixed first mass of 200 m/z after accumulation of 3e^6^ ions or after filling time of 35 ms (whichever occurred first). Data was acquired in profile mode. For data acquisition and processing of the raw data XCalibur 4.0 (Thermo Scientific) and Tune version 2.9 were employed. For sample-specific spectral library generation, data was acquired from samples from each condition in data-dependent acquisition (DDA) mode, using the same gradients as the DIA analyses. Both DIA and DDA data were included in the library generation. The data were searched against the human Uniprot database using the Pulsar search engine (Biognosys AG). The following modifications were included in the search: Carbamidomethyl (C) (Fixed) and Oxidation (M)/Acetyl (Protein N-term) (Variable). A maximum of 2 missed cleavages for trypsin were allowed. The identifications were filtered to satisfy FDR of 1% on peptide and protein level. The resulting library contained 99,274 precursors corresponding to 6334 protein groups. Precursor matching, protein inference, and quantification were performed in Spectronaut using median peptide and precursors (no TopN). Relative quantification was performed in Spectronaut (version 13.1.190621, Biognosys AG) using the paired samples (according to the day of cell harvesting) from each condition across the replicates. The data (candidate table) and data reports (protein quantities) were then exported and further data analyses and visualization were performed with R-studio (version 0.99.902) using in-house pipelines and scripts. Proteome data sets were further processed using ingenuity pathway analysis (IPA; Quiagen) (cutoff of *q* < 0.05).

### Statistical analysis

Statistical analyses were done with Microsoft Excel 2016 using a two-tailed Student's *t* test or GraphPad 8 using two-way ANOVA tests (*p < 0.05, **p < 0.01, ***p < 0.001). Error bars show standard error of the mean (SEM) if not mentioned otherwise.

## Results

### Apoptosis induction in ES cells after inhibitor combinations

**Fig. 1:**
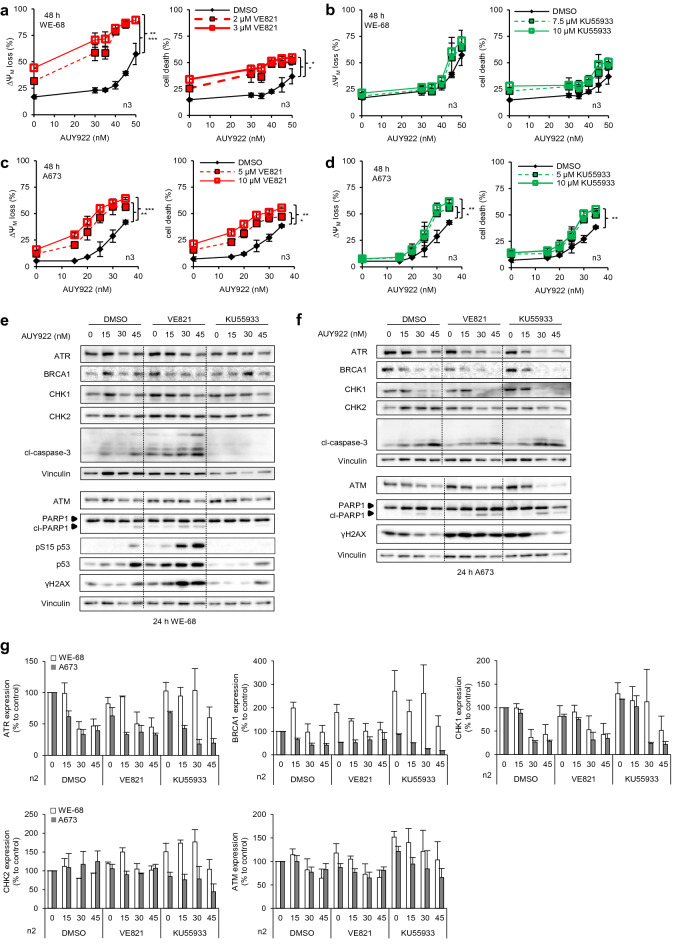
Increased apoptosis after ATRi and HSP90i combinations. p53 wild-type WE-68 and p53 null A673 cells were treated with the indicated concentrations of AUY922, VE821, KU55933 and their combinations. DMSO was used as control. **a**–**d** The loss of the mitochondrial transmembrane potential (ΔΨ_M_) and cell death were assessed by flow cytometry after 48 h. All graphs show the mean ± SEM of three independent experiments. Statistical analysis was done using two-way ANOVA test (*p < 0.05; **p < 0.01; ***p < 0.001). WE-68 (**e**) and A673 (**f**) cells were treated with 15–45 nM of AUY922, 2 µM of VE821, 5 µM of KU55933 and their combinations. Analysis of indicated proteins was done by Western blot after 24 h; vinculin was used to control protein loading. Immunoblots are representative of at least two independent experiments. Quantification of selected proteins is shown in **g**

In our study we asked if ATRi would increase the effectiveness of HSP90 inhibition in ES cells. To study p53-dependent effects, we used p53^wt^ WE-68 and p53^−/−^ A673 cells (Additional file 1: Fig. S1A) and treated them with AUY922 and VE821. For comparison, we also applied the ATM inhibitor (ATMi) KU55933 to study the role of different DDR pathways upon HSP90i treatment. As readouts, we measured the loss of the mitochondrial transmembrane potential (ΔΨ_M_), a marker for apoptosis [18, 20], and cell death by flow cytometry. Low nanomolar concentrations (10–50 nM) of AUY922 induced ΔΨ_M_ loss and cell death in both ES cell lines (Fig. 1a–d). The combinations with either VE821 (AUY-VE) or KU55933 (AUY-KU) enhanced the cytotoxicity of AUY922 especially at low concentrations, with a stronger effect of AUY-VE (Fig. 1a–d). Two-way ANOVA analysis revealed that AUY-VE significantly augmented the loss of ΔΨ_M_ (*p* < 0.01 or lower) and cell death (*p* < 0.05 or lower) in both ES cell lines, whereas AUY-KU showed only in A673 statistically significant effects (*p* < 0.05 or lower). Similar observations were made in p53^−/−^ SaOS-2 osteosarcoma cells (Additional file 1: Fig. S1B-C). This demonstrated the potential of AUY-VE as an effective treatment for both p53^wt^ and p53^−/−^ cells. Nevertheless, the ΔΨ_M_ decay reached 90% in WE-68 cells, but only 64% in A673 and 71% in SaOS-2 cells after AUY-VE (compare Fig. 1a, c and Additional file 1: Fig. S1B), suggesting a p53-dependent induction of mitochondrial-mediated apoptosis in p53^wt^ cells.

### Amplification of AUY922-mediated cellular damages by AUY-VE but not by AUY-KU

Next, we delineated the molecular basis of AUY-VE-mediated cytotoxicity in ES cells. We investigated which concentrations of VE821 and KU55933 were sufficient to fully inhibit ATR- or ATM-mediated signaling. The activation of members from both DDR pathways can be judged by their phosphorylation [21]. Western blot analysis showed that 30 nM AUY922 abolished the phosphorylation of ATR and CHK1 in both ES cell lines, accompanied by a marked decrease of ATR and CHK1 total protein level (Additional file 1: Fig. S1E, F). In contrast, ATM phosphorylation and protein level were not affected by AUY922. All applied concentrations of AUY-VE further augmented these effects and impaired ATR signaling but activated ATM in parallel. AUY-KU impaired ATM phosphorylation at all tested concentrations but stabilized ATR protein level. The protein level and activation of CHK2, typically indicated by a super-shift of the CHK2 main protein band [22], was not affected by any of the applied treatments (Additional file 1: Fig. S1E, F). Thus, even low concentrations of AUY-VE or AUY-KU were sufficient to inhibit the corresponding DDR signaling in ES cells.

Based on these findings and the observation that high concentration of VE821 alone were cytotoxic to WE-68 cells (Fig. 1a), we decided to use 2 µM of VE821 and 5 µM KU55933 in both cell lines in the subsequent experiments to allow for a comparative molecular analysis of p53-dependent effects of AUY-VE and AUY-KU. Since HSP90i are known to induce DNA damage [23], we analyzed the expression level of key DDR molecules, together with DNA damage markers by Western blot. AUY922 and AUY-VE strongly reduced the expression of ATR and CHK1 and mildly reduced that of ATM, while they did not affect CHK2 protein levels (Fig. 1e-g). Interestingly, BRCA1 expression showed cell line-specific responses to the treatments: it was decreased in A673 cells and increased in WE-68 cells (particularly obvious at high concentrations). AUY-KU, however, increased the abundance of all studied DDR proteins in WE-68 cells and of ATM and CHK1 in A673 cells.

In addition, AUY922 and AUY-VE induced signs of apoptosis and DNA damage. High concentrations of AUY922 induced the expression of p53, phosphorylation of p53 at its serine 15 residue (pS15-p53) and of H2AX (γH2AX), both signs of active DDR [24], as well as the cleavage of PARP1 (cl-PARP1) and caspase-3 (cl-caspase-3) in WE-68 cells, indicative of ongoing apoptosis [15] (Fig. 1e). AUY-VE strongly increased the level of γH2AX, cl-caspase-3 and cl-PARP1, p53 and p-p53. In contrast, AUY-KU abolished their presence, suggesting a protective function of KU55933 (Fig. 1e). In A673 cells, AUY922 also induced cleavage of caspase-3 and PARP1 but to a lower extent (comparing Fig. 1e and f). Surprisingly, the level of γH2AX was decreased at high concentrations of AUY922, likely due to a stronger depletion of DDR proteins in A673 cells that impaired DNA damage recognition. AUY-VE and AUY-KU enhanced the level of γH2AX and cl-PARP1, but not of cl-caspase-3, whereas the effects of AUY-VE were stronger and more persistent (Fig. 1f).

### Increased treatment effectiveness of AUY-VE in p53 wild-type ES cells

**Fig. 2:**
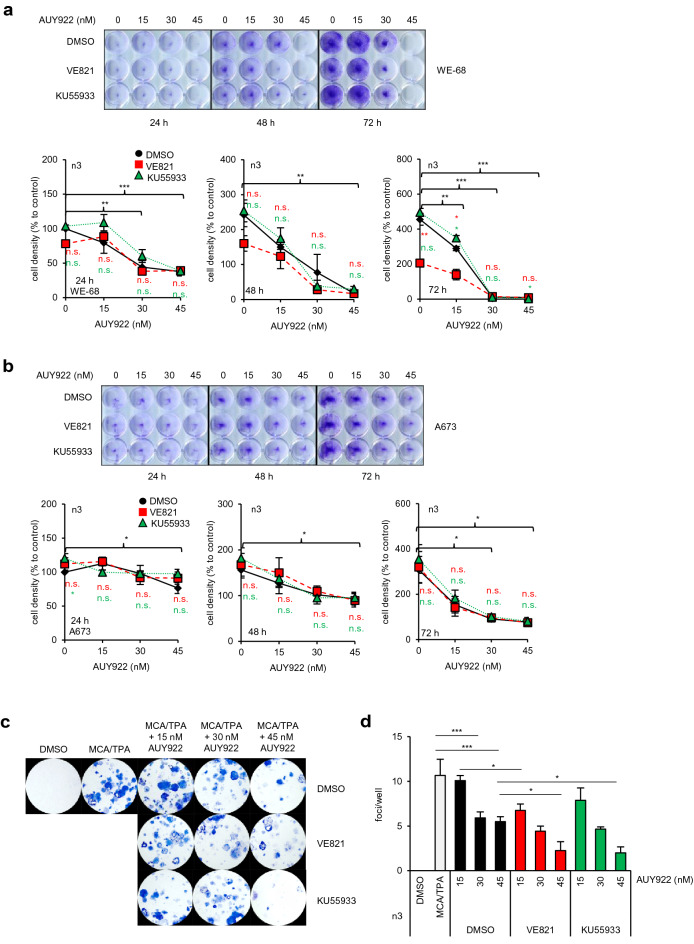
Decreased cell proliferation after AUY-VE in WE-68 cells. WE-68 (**a**) and A673 (**b**) cells were treated with 15–45 nM of AUY922, 2 µM of VE821, 5 µM of KU55933 and their combinations. Cell densities at 24, 48 and 72 h after treatment were assessed by crystal violet-based cell proliferation assay. DMSO was used as control. **c** Representative images of a BALB/c cell transformation assay (BALB-CTA) after 72 h of treatment with 15–45 nM AUY922 ± 2 µM VE821 or 5 µM KU55933. MCA/TPA-transformed cell foci are Giemsa stained and appear in blue. Quantification of the number of type-III foci/well from BALB-CTA experiments is shown in D. All graphs show the mean ± SEM of three independent experiments (*p < 0.05; **p < 0.01; ***p < 0.001)

This led us to study whether the effects of the drug treatment were reflected in cell proliferation using a crystal violet-based assay. AUY922 decreased cell proliferation in a concentration- and time-dependent fashion. Already after 24 h, cell densities were significantly reduced by 45 nM AUY922, with stronger effects being observed in WE-68 (61%; *p* < 0.001) than in A673 cells (23%; *p* < 0.05) (Fig. 2a, b). The effects were even more pronounced after 48 h. Cell densities of WE-68 cells went close to zero after 72 h treatment with 30–45 nM AUY922 (*p* < 0.001), whereas cell densities of A673 cells after 72 h were just reduced about 20% compared to their initial control value at 24 h and remained unchanged thereafter (Fig. 2b). AUY-VE was again more effective than AUY922 alone in WE-68 cells after 48 and 72 h treatment, whereas AUY-KU had no additional effect (Fig. 2a). To our surprise, we did not find differences in cell densities of A673 cells at any time point after AUY-VE or AUY-KU compared to AUY922 alone (Fig. 2b).

To investigate if higher ATRi concentrations were necessary to impair the proliferation of A673 cells, we combined 5 µM VE821 with AUY922 and analyzed cell densities afterwards. Consistent with our initial experiments (Fig. 1a–d), we found that 5 µM VE821 itself was cytotoxic to WE-68 cells and greatly reduced their proliferation, with the strongest effect being observed after 72 h of treatment (Additional file 2: Fig. S2A). Thus, AUY-VE combinations were hardly more effective than VE821 alone. We did not observe any differences in cell densities between 2 and 5 µM VE821 alone or in combination with AUY922 in A673 cells (compare Fig. 2b and Additional file 1: Fig. S2B). Hence, 2 µM proofed to be the most suitable concentration of VE821 for our study.

We also tested if the treatments elicited cell cycle arrest in ES cells, however, we did not detect any prominent alterations of cell cycle profiles after 24 and 48 h of treatment (Additional file 3: Fig. S3). Taken together, AUY922 and AUY-VE effectively reduced the proliferation of ES cells; the weaker effects observed in A673 cells were likely the result of lower apoptotic activity due to p53 deficiency.

### AUY-VE is suitable to treat other types of cancer beyond ES

To analyze if our observations could be generalized or were limited to ES cells, we used isogenic p53^wt^ and p53^−/−^ HCT116 colon carcinoma cells and analyzed them by Western blot (Additional file 4: Fig. S4A). AUY922 decreased the expression of ATR, ATM and BRAC1 independent of the p53 status. VE821 and AUY-VE had no effect on ATM stability but further reduced the levels of BRCA1 and ATR. HCT116 p53^wt^ cells had more cl-caspase-3 after AUY922 treatment, which was further increased after AUY-VE, whereas p53^−/−^ cells had less cl-caspase-3, despite more DNA damage, as judged by γH2AX abundance. Similarly, the level of cl-PARP1 showed a stronger increase in p53^wt^ than in p53^−/−^ cells after AUY922 treatment and AUY-VE. Overall, we found similar protein alterations in both ES and HCT116 cells (compare Fig. 1e, f and Additional file 4: Fig. S4A) by AUY922 and AUY-VE, with stronger effects in ES cells.

Furthermore, we performed a BALB/c cell transformation assay (BALB-CTA) [16] to study the therapeutic potential of AUY-VE in comparison to AUY-KU (Fig. 2c, d and Additional file 4: Fig. S4B). Malignant transformation of BALB/c cells was induced via consecutive MCA and TPA treatments. 30–45 nM AUY922 significantly reduced the number of type-III foci (both *p* < 0.001; Fig. 2d). AUY-VE led to a further reduction even at a low concentration of 15 nM AUY922 (*p* < 0.05). AUY-KU was less effective at low concentrations, but similarly effective as AUY-VE at 45 nM AUY922 (*p* < 0.05 to AUY922). Thus, AUY-VE showed therapeutic potential already at low concentrations and in different cell models beyond ES cells independent of their p53 status.

### Activation of p53 target gene transcription in WE-68 cells after AUY-VE

**Fig. 3:**
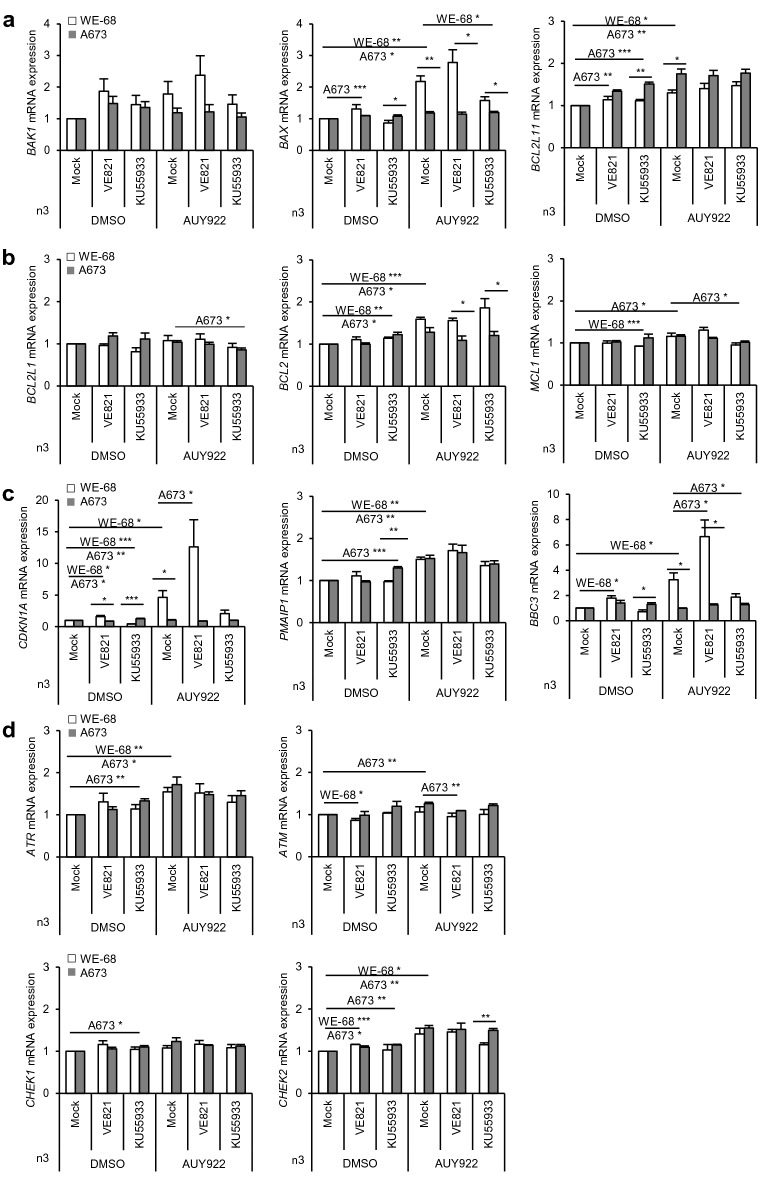
Increased p53 target gene expression in WE-68 cells. WE-68 cells were treated with 30 nM of AUY922, 1 µM of VE821, 7.5 µM of KU55933 and their combinations; A673 cells were treated with 15 nM of AUY922, 1 µM of VE821, 5 µM of KU55933 and their combinations. DMSO was used as control. **a**–**d** The mRNA expression of indicated genes was analyzed after 24 h by qPCR. All graphs show the mean ± SEM of three independent experiments (*p < 0.05; **p < 0.01; ***p < 0.001)

To gain further molecular insight, we analyzed mRNA expression by qPCR (Fig. 3) using effective doses of the inhibitors found in the preceding experiments (Figs. 1 and 2). In WE-68 cells, we observed an upregulation of pro-apoptotic *BAK1*, *BAX* (*p* < 0.01) and *BCL2L11* (BIM) (*p* < 0.05) after AUY922 treatment, with the p53 target gene *BAX* [25] showing the strongest effects (Fig. 3a). AUY-VE further increased while AUY-KU decreased these mRNA levels. In A673 cells, we found no change of *BAK1* and *BAX* but an increase in *BCL2L11* expression after AUY922 treatment (*p* < 0.01), which was not affected by AUY-VE or AUY-KU. Among anti-apoptotic BCL2 family members, *BCL2L1* (BCL-XL) and *MCL1* mRNAs remained unchanged in both ES cell lines (Fig. 3b). *BCL2* was slightly increased after AUY922 treatment in WE-68 (*p* < 0.001) but not in A673 cells. Since we discovered recently an important function of AIF in p53 null ES cells [18], we also determined *AIFM1* mRNA expression after AUY922 treatment (Additional file 4: Fig. S4C). We found *AIFM1* expression to be only mildly elevated (*p* < 0.05 for A673), with no further increase by AUY-VE or AUY-KU. Thus, AIF seemed to be not involved in apoptosis induction after AUY-VE.

Upon analyzing additional p53 target genes, we found that AUY922 increased the mRNA expression of *CDKN1A* (p21) and pro-apoptotic *BBC3* (PUMA) [25] (*p* < 0.05 for both), which were both greatly further enhanced by AUY-VE in WE-68 cells (Fig. 3C). In contrast, AUY-KU did not change *CDKN1A* and *BBC3* mRNAs levels. The mRNA of pro-apoptotic *PMAIP1* (NOXA) [25] was increased after AUY922 treatment (*p* < 0.01 in both ES cell lines) and remained unchanged after AUY-KU and AUY-VE. Further, we found a small increase in *TP53* expression after AUY922 in WE-68 cells (*p* < 0.01), without an additional effect of AUY-VE or AUY-KU (Additional file 4: Fig. S4D). In contrast, *MDM2* levels were significantly increased after AUY922 (*p* < 0.01), further enhanced by AUY-VE, but decreased by AUY-KU (Additional file 4: Fig. S4D).

Since the treatments had affected the expression of DDR proteins (Fig. 1e, f), we also investigated the mRNA levels of *ATR*, *ATM*, *CHEK1* and *CHEK2* and found that *ATM* and *CHEK1* expressions were not impaired by any treatment (Fig. 3d). The expression of *ATR* (*p* < 0.05 for A673; *p* < 0.01 for WE-68) and *CHEK2* (*p* < 0.01 for A673; *p* < 0.05 for WE-68) was increased by AUY922 but not affected by AUY-VE or AUY-KU. These results suggest that the decay of DDR proteins (see Fig. 1e, f ) was not the result of altered gene expression, but rather of reduced protein stabilities. Taken together, the upregulation of pro-apoptotic p53 target gene expression by AUY922 that is further enhanced by AUY-VE may explain the strong induction of apoptosis in p53^wt^ WE-68 cells (Fig. 1a–d).

### Pro-apoptotic ER stress occurs in A673 cells after AUY-VE

**Fig. 4:**
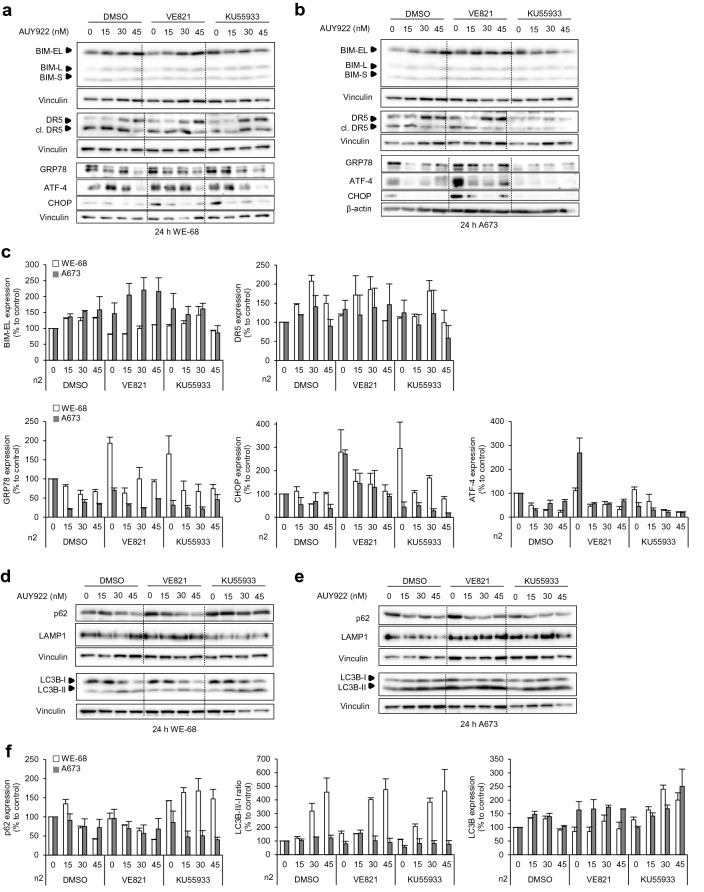
Increased autophagy and ER stress in A673 cells. WE-68 and A673 cells were treated with 15–45 nM of AUY922, 2 µM of VE821, 5 µM of KU55933 and their combinations. DMSO was used for control. **a**, **b**, **d**, **e** Analysis of indicated proteins was done by Western blot after 24 h; vinculin and β-actin were used to control protein loading. Immunoblots are representative of at least two independent experiments. **c**, **f** Quantifications of selected proteins

HSP90i induce an unfolded protein response (UPR) and endoplasmic reticulum (ER) stress [26, 27]. We speculated that A673 cells might suffer from higher level of ER stress due to their lack of p53-mediated pro-apoptotic mechanisms. In both ES cell lines, BIM and DR5 accumulated in response to AUY922 (Fig. 4a–c), which reflect ER stress [28, 29]. Western blot analysis revealed that AUY-VE had no additional effect on DR5, but further increased BIM levels particularly in A673 cells and reduced them in WE-68 cells. AUY-KU did not affect BIM levels but decreased the expression of DR5 in both ES cell lines (Fig. 4a–c ). The accumulation of the HSP GRP78 in response to unfolded proteins is another characteristic of ER stress, which can lead to ATF-4 and CHOP induction to mediate pro-apoptotic ER stress responses after severe or persistent stress [30–32]. We found that VE821 greatly induced the expression of ATF-4 and CHOP in A673 cells (Fig. 4a–c). In contrast, we observed that VE821 as well as KU55933 induced the expression of GRP78 and CHOP in WE-68 cells, indicating an adaptive response to ER stress in these cells [30–32].

Flow cytometry showed that reactive oxygen species (ROS), another sign of ER stress [33], were decreased in WE-68 cells after AUY922 treatment (Additional file 5: Fig. S5A). In contrast, they accumulated in A673 cells and reached a plateau at 30–45 nM AUY922. The amount of ROS, however, was not further enhanced by AUY-VE or AUY-KU. Altogether, these data show that both AUY922 and VE821 induced ER stress in A673 cells, and that AUY-VE increased the level of pro-apoptotic ER stress.

To study the autophagy response as a result of ER stress, we analyzed LC3B, p62 and LAMP1 expressions [30, 31, 34] (Fig. 4D-F and Additional file 5: Fig. S5B). p62 decreased in both ES cell lines in response to AUY922 and AUY-VE. AUY-KU increased the p62 level in WE-68 cells but had no additional effect in A673 cells compared to AUY922. LAMP1 was decreased after AUY922, which however was stabilized by AUY-VE in both ES cell lines. The total amount of LC3B was only mildly affected by AUY922 or AUY-VE in WE-68 cells, whereas more LC3B-I converted to LC3B-II after both treatments, indicating ongoing autophagy [34]. In A673 cells, the total LC3B level was increased by AUY-VE, indicative of stronger autophagy in these cells, whereas none of the treatments changed the LC3B-II/I ratio.

We used again isogenic p53^wt^ and p53^−/−^ HCT116 cells to investigate if our observations in ES cells could be generalized. Western blot analysis showed that the HCT116 cell lines increased the expression of LC3B, BIM and DR5 in response to AUY922, VE821 and their combination in a comparable manner (Additional file 5: Fig. S5C). The increase in BIM, however, was stronger in p53^−/−^ HCT116 cells. Moreover, AUY-VE specifically increased ATF-4 and LAMP1 protein level in p53^−/−^ HCT116 cells, indicating higher level of ER stress and autophagy in p53-deficient cells after AUY-VE, thus validating our observations in ES cells.

In sum, AUY922 induced pro-apoptotic ER stress and autophagy in ES cells, and AUY-VE particularly enhanced them in A673 cells. AUY-KU showed again protective effects and reduced ER stress marker in both ES cell lines.

### AUY-VE causes severe intracellular damages

**Fig. 5:**
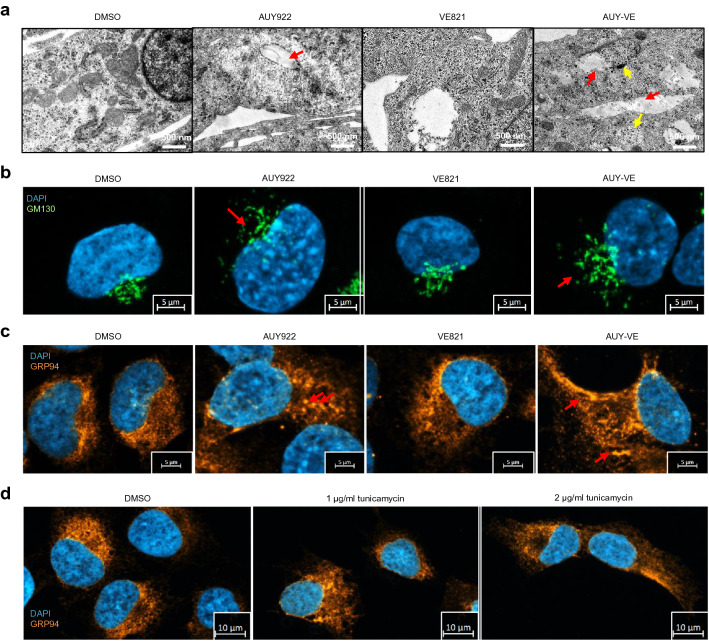
Increased intracellular damages after AUY-VE. A673 cells were treated with 45 nM AUY922 ± 2 µM VE821 for 24 h. **a** Intracellular structures were analyzed by transmission electron microscopy (TEM). Red arrows indicate lipid-filled vesicles; yellow arrows indicate lysosomes. **b** Golgi networks were analyzed by fluorescence microscopy of GM130. Red arrows indicate dispersed Golgi structures. **c** Endoplasmic reticulum (ER) networks were analyzed by fluorescence microscopy of GRP94. Red arrows indicate vesicular ER structures. A673 cells were treated with 1–2 µg/ml of tunicamycin for 24 h. **e** ER networks were analyzed by fluorescence microscopy of GRP94. **c**–**e** DAPI was used to stain cell nuclei. A shows one representative experiments; B-E are representative for at least two independent experiments

To visualize intracellular defects, we performed transmission electron microscopy (TEM) of WE-68 and A673 cells (Fig. 5A, Additional file 6: Fig. S6 and Additional file 7: Fig. S7a–d). We observed that AUY922 and VE821 led to similar defects in both ES cell lines, i.e., an accumulation of autophagosomes, lysosomes, big and empty late lysosomes, β-glycogen granules, aberrant mitochondria and ER structures as well as lipid droplets and lipid-filled vesicular structures. In some cells, we found ER-derived multi-lammelar structures (Additional file 6: Fig. S6 and Additional file 7: Fig. S7a–d). AUY-VE further enhanced the defects leading to an accumulation of different vesicles, sick mitochondria and ER structures as well as to massive lipid-filled vesicles, originating from lysosomal structures. We found mitochondria and ER structures within autophagosomes, which were partially secreted by the cells (Additional file 6: Fig. S6 and Additional file 7: Fig. S7a–d). In any case, we noted a higher degree of cell ruptures, indicative for late stages of apoptosis [35], in WE-68 cells after treatment but stronger accumulation of vesicles and aberrant cell organelles in A673 cells, confirming stronger ER stress and autophagy in the latter.

Next, we analyzed Golgi and ER structures by immunofluorescence microscopy in A673 cells. The fragmentation of the Golgi apparatus can be a result of high ER stress level [30, 32]. We visualized Golgi networks by immunostaining the Golgi marker GM130 [36] and found that AUY922 and particularly AUY-VE induced the fragmentation of Golgi structures, whereas VE821 alone had no such effect (Fig. 5B). To analyze ER networks, we immunostained the ER-resident HSP GRP94 [36]. AUY922 induced ER structural defects, characterized by an accumulation of vesicular structures and slight dispersal of the ER network (Fig. 5C). VE821, however, induced a more dispersed ER network and AUY-VE induced a combination of both defects to a higher degree. Tunicamycin is a known ER stress inducer, which inhibits N-linked protein glycosylation within the ER [37]. We observed by Western blot that even low concentrations of tunicamycin induced a strong accumulation of the ER stress markers GRP78 and CHOP in A673 cells (Additional file 7: Fig. S7E). In fact, the ER defects after VE821 treatment were similar to those after tunicamcyin treatment (compare Fig. 5C and 5D), indicating a prominent and previously unrecognized function of ATR at the ER. Taken together, AUY922 and VE821 induce ER stress and intracellular damages in a p53-independet manner. AUY-VE increases these defects and further augments ER stress level in p53-deficient ES cells.

### Proteome analysis of A673 cells validates ER stress induction after AUY-VE

**Fig. 6:**
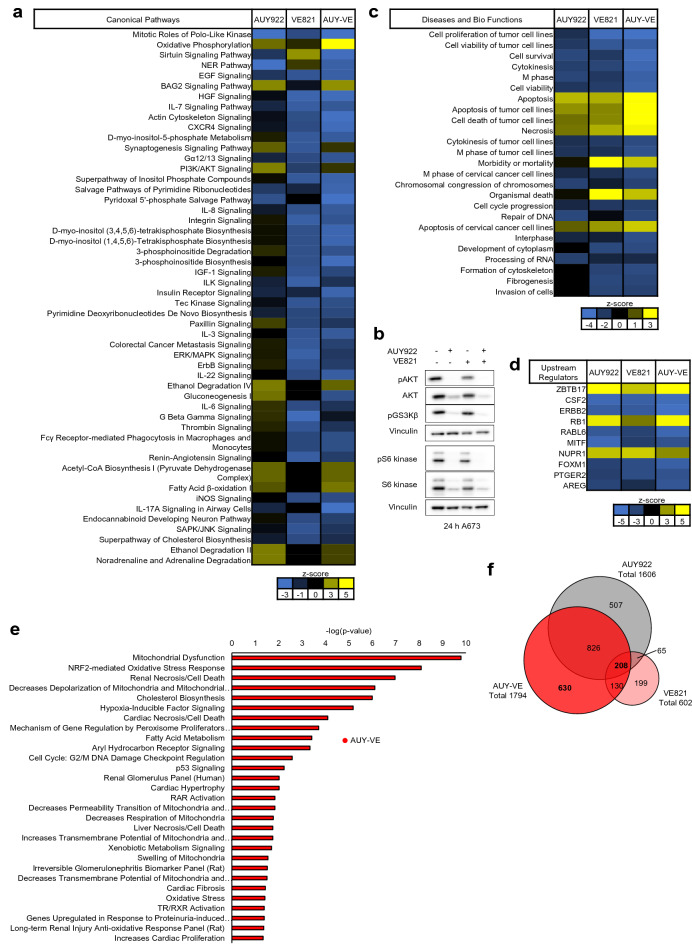
ATRi and HSP90i share common molecular targets. A673 cells were treated with 45 nM AUY922 ± 2 µM VE821 for 24 h and a quantitative whole proteome analysis was done by mass spectrometry from three individual experiments (refer to Additional file 11: File S1). The proteome data set was further processed by ingenuity pathway analysis (IPA; cutoff: *q* < 0.05) for alterations among canonical pathways (**a**), diseases and bio functions (**c**) and upstream regulators (**d**). The activation z-score of individual processes/molecules is shown in yellow for activation and blue for inactivation. **b** Analysis of indicated proteins was done by Western blot; vinculin was used to control protein loading. Immunoblots are representative of at least two independent experiments. **e** IPA of toxicities after AUY922 in combination with VE821 (AUY-VE). **f** DEPs of individual treatments (cutoff: *q* < 0.05) and their overlaps

Finally, we performed quantitative mass spectrometry analysis of the whole A673 cell proteome to validate our findings (Fig. 6, Additional file 8: Fig. S8, Additional file 10: Fig. S9; for individual protein changes refer to Additional file 11: File S1). AUY922 and AUY-VE induced the strongest proteome alterations (Additional file 8: Fig. S8A-C and Additional file 11: File S1), which was also reflected by PCA analysis (Additional file 8: Fig. S8D). Ingenuity pathway analysis (IPA) of the data sets (cutoff: *q* < 0.05; log2-fold change > 0.5) (Fig. 6A, 6C-E and Fig. Additional file 9: S9A-B) showed that both AUY922 and AUY-VE decreased DNA maintenance pathways alongside to growth factor signaling and cytoskeletal organization but activated the chaperone regulator BAG2 and PI3K/AKT signaling (Fig. 6A). Western blot analysis of the PI3K/AKT/mTOR signaling pathways in A673, WE-68 and HCT116 cells showed that AUY922 and AUY-VE greatly reduced the expression level of phospho-GSK3β, AKT and p70 S6 kinase together with their phosphorylation in all tested cell lines, yet strongest effects were observed in ES cells (Fig. 6B and Additional file 1: Fig. S9A-B). VE821 particularly reduced PI3K/AKT/mTOR signaling in p53-deficient A673 cells (Fig. 6B). In addition, IPA analysis of the proteomics data showed that all treatments reduced cell viability and proliferation of A673 cells and activated cell death and apoptosis (Fig. 6C), with the strongest effect by AUY-VE. These pathway alterations were regulated by proteins involved in cell cycle regulation and proliferation, inflammation, DNA damage recognition, cancer progression and stress response [38–43] (Fig. 6D). IPA further predicts strong mitochondrial dysfunctions, oxidative stress and changes of fatty acid (FA) metabolism after AUY922 and VE821 single treatments (Additional file 10: Fig. S10A-B) and most prominently after AUY-VE (Fig. 6E).

Moreover, we found an overlap of 208 proteins among the differentially expressed proteins (DEPs; cutoff: *q* < 0.05) of all three treatments, which were altered in a similar fashion (Fig. 6F and Additional file 11: File S1). IPA showed that they are involved in apoptosis and cell proliferation, FA and cholesterol metabolism (Additional file 10: Fig. S10C-D). In parallel, we found 630 DEPs (cutoff: *q* < 0.05) which were unique for AUY-VE (Fig. 6F and Additional file 11: File S1), and IPA showed that they were involved in reduced cell survival, cytoskeletal organization, DNA maintenance, increased cell death, oxidative stress and mitochondrial dysfunctions (Additional file 10: Fig. S10E-F).

These findings indicate that AUY922, VE821 and their combination share common molecular targets, which mediate the synergism between both compounds. In correlation with our initial experiment (Fig. 1), AUY-VE further promoted cell death in A673 cells by impairing PI3K/AKT/mTOR-mediated signaling and by increasing mitochondrial dysfunctions and DNA damage.

## Discussion

In our study, we show that the combination of ATRi and HSP90i amplified their individual effects. AUY922 induced DNA and mitochondrial damage, ER stress and autophagy in both tested ES cell lines, whereas the combination with VE821 increased apoptosis based on two distinct mechanisms. KU55933, however, had rather protective effects. Although ATM and ATR share common cytosolic phosphorylation targets [44], their functions upon HSP90 inhibition seem to be opposed. ATR is activated by SSBs and replicative stress (RS) to maintain genomic stability [14, 44, 45]. ES cells, which are deficient in BRCA1-mediated HR, suffer from permanent RS, rely on ATR-CHK1 pathways and are thus sensitive to ATRi [1, 4, 7, 10, 12]. In addition, we found that both ATRi and HSP90i share a set of common molecular targets, involved in cell vitality and proliferation and ER stress response. Hence, AUY-VE greatly increased the apoptosis and cell death compared to AUY922 in ES cells.

In p53^wt^ WE-68 cells, AUY-VE enhanced the amount of DNA damage and p-p53 levels compared to AUY922, leading to pro-apoptotic p53 target gene expression and mitochondria-mediated apoptosis, reflected by high level of ΔΨ_M_ loss, cl-caspase-3 and cl-PARP1. The ATM-mediated phosphorylation of p53 [45, 46] after AUY922 and AUY-VE treatments seemed to be necessary to induce the pro-apoptotic gene expression in WE-68 cells. AUY-KU abolished the phosphorylation/activation of p53 and impaired its pro-apoptotic target gene transcription, which protected WE-68 cells from pronounced apoptosis.

A673 cells, lacking p53, had lower levels of apoptosis and their proliferation was less impaired by AUY922, validating that p53 null/mutant ES cells are more therapy resistant [5, 6]. AUY-VE also increased the apoptosis in A673 cells resulting from high level of ER stress, autophagy, strongly reduced DDR proteins and, in turn, unrepaired DNA damage. Contrary to this, AUY-KU stabilized the expression of DDR proteins in both ES cell lines and reduced ER stress marker in A673 cells compared to AUY922. This may explain the low combined effectiveness of AUY-KU compared to AUY-VE.

We further noted that VE821 and AUY-VE impaired ATR signaling but activated ATM, which was connected to high ER stress in all tested cells lines. In contrast, KU55933 and AUY-KU inhibited ATM but stabilized proteins from ATR signaling pathways and reduced ER stress level. This indicates that both PIKK family members have different functions for ER maintenance, with functional ATR protecting cells from ER stress.

In the following, we will discuss single aspects of our study in detail. ATR, CHK1, DNA-PK, FANCA and BRCA2 are known HSP90 clients and HSP90i decrease their abundance [47, 48]. In both ES cell lines, AUY-VE increased γH2AX level, which decreased at higher concentrations in A673 cells, suggesting an accumulation of unrecognized DNA damages linked to depleted DDR proteins. We found by Western blot and proteome analysis that AUY922 reduced the abundance of several DDR proteins, namely ATM, ATR, CHK1, BRCA1, ERCC and FANC proteins, 53BP1, RAD proteins, WRN, XRCC (for details refer to Fig. 1E-G and Additional file 11: File S1), expanding the list of known HSP90 clients from the DDR. AUY-VE further decreased their levels with strongest effects in A673 cells. The stronger accumulation of γH2AX and depletion of DDR proteins after AUY-VE in p53^−/−^ cells was confirmed in HCT116 cells and might be a result of high ER stress and autophagy.

HSP90 family proteins are located within the cytosol, nucleoplasm, ER and mitochondria and are responsible for folding, stability, activation and turnover of several client proteins [13]. Thus, HSP90i induce unfolded protein response (UPR) and ER stress leading to an accumulation of chaperones and ER structures [26, 28, 49] and to apoptosis in severe cases [26, 27, 50–52]. In line with this, proteome analysis revealed an overexpression of ER, Golgi and lysosomal/endosomal proteins together with disturbed ER and Golgi networks seen by fluorescence microcopy in A673 cells after AUY-VE treatment, indicating severe ER stress in these cells. BIM, PUMA, BAX and BAK are important to mediate ER stress-induced mitochondrial disintegration and apoptosis; DR5 and caspase-8 are involved, but not essential [26–29, 49]. We observed an accumulation of BIM and DR5 in response to AUY922 in ES cells. BIM protein and mRNA levels were both enhanced by AUY-VE, specifically in A673 cells. Hence, BIM in concert with DR5 seems to play an important function for ER stress-mediated apoptosis in these cells. Pro-apoptotic PUMA, BAX and BAK mRNAs increased only in WE-68 cells, which showed less sign of ER stress, but of mitochondrial-mediated apoptosis after AUY-VE treatment. p53 target genes are involved in cell cycle arrest, DNA repair, apoptosis, metabolism, autophagy and mRNA translation [24, 25]. Dependent on the type, duration and level of stress, p53 mediates either repair or cell death functions [46, 53]. It is thus plausible that VE821 and the associated higher degree of DNA damage changed the cell fate decision determined by p53 and induced the intrinsic apoptosis pathway in WE-68 cells.

Lipid droplets (LD) store FAs and sterols, originate from the ER or mitochondria and are connected to ER stress and apoptosis [54, 55]. Autophagosomes also originate from the ER after ULK1, PI3K and ATG protein recruitment [34]. We found by proteome analysis increased proteins from carbohydrate and FA metabolism after AUY-VE in A673 cells. TEM pictures validated the accumulation of vesicular structures, which were partially lipid or glycogen filled alongside to mitochondrial and ER damages. GSK3 controls glycogen metabolism and inflammatory cytokines and is regulated by PI3K/AKT/mTOR signaling [56, 57]. Alterations of PI3K/AKT/mTOR signaling can be caused by UPR activation [58]. The corresponding proteins were decreased in A673 cells together with JAK/STAT proteins, which are activated by cytokines, as observed by our proteomic screen after AUY-VE (for details refer to Additional file 11: File S1). In addition, we found greatly impaired PI3K/AKT/mTOR signaling after AUY-VE in ES and HCT116 cells by Western blot. Thus, AUY-VE induced metabolic and inflammatory signaling alterations via the PI3K/AKT/mTOR axis leading to a toxic accumulation of FA-, lipid- or glycogen-filled vesicles in ES cells.

The HSP90i geldanamycin induces autophagy via inhibition of AKT/mTOR signaling in osteosarcoma cells [52]. Although we found an increased expression of LC3B, elevated level of mTOR and LAMTOR after AUY-VE treatment, AKT, p70 S6 kinase, p62, ATG family and RICTOR protein levels were decreased (for details refer to Fig. 4A-B, 6B, Additional file 1: Fig. S10 and Additional file 11: File S1). Thus, mTORC2 and macroautophagy seemed to be impaired, whereas lysosomal/endosomal processes were activated [59]. Chaperone-mediated autophagy (CMA) involves HSP70, LAMP2 and lysosomes to regulate cell metabolism, inflammation and the cell cycle in response to stress [34, 60]. We found HSP70 family proteins and LAMP1 upregulated in A673 cells in the proteome and Western blot analyses, indicating CMA after AUY-VE treatment. BAG2 signaling was predicted to be active after AUY-VE, although its protein level was decreased, according to the proteome analysis. BAG2 interacts with HSP70 to regulate its functions and suppresses apoptosis induction via BCL2 protein interactions [61, 62]. Thus, reduced BAG2 and high HSP70 levels might contribute to CMA and apoptosis induction in A673 cells.

ES cells have an aberrant activity of AKT, (p)ERK, MYC, HIF1α, VEGF and IGF proteins [1], which can all be decreased by HSP90i [13, 26, 27, 63]. This was confirmed by our proteome analysis. PI3K/AKT signaling and high SIRT1 expression is associated with metastasis of ES cells [1]. During metastasis, an epithelial-to-mesenchymal transition is described for a subpopulation of ES cells, which involves the reorganization of the cytoskeleton [1]. We found reduced levels of SIRT1, SIRT2, AKT and PI3K subunits together with several cytoskeletal proteins by the proteome analysis in A673 cells as well as impaired PI3K/AKT/mTOR signaling by Western blot in both ES cell lines after AUY-VE. The fusion protein EWS-FLI1 suppresses wild-type EWSR1 functions to activate RNAPII and transcription [1, 4, 64]. BCOR and epigenetic modifiers are involved in the transcriptional regulation of ES cells [1, 64]. We found BCOR and several epigenetic modifiers, including KATs/KDACs and KDMs/KMTs downregulated after AUY-VE (for details refer to Additional file 11: File S1), potentially thwarting EWS-FLI1 activities. In addition, sirtuin signaling was predicted to be inactive by IPA. SIRT3 was the only exception accumulating after AUY-VE treatment (Additional file 11: File S1). SIRT3 expression is induced by ER, metabolic or oxidative stress and activates metabolic adaptations and autophagy [58, 65, 66], which corresponds to our observations in A673 cells. In addition, we found repressed ERBB2 (HER2) and FOXM1 signaling by IPA, both of which are involved in tumor progression and metastasis [42, 43]. Thus, AUY-VE might be suitable for the treatment of aggressive/metastatic ES tumors irrespective of their p53 status.

## Conclusions

This study establishes a novel anti-cancer strategy to treat ES cells by combination of low-dosed HSP90i and ATRi, which proved to be highly effective irrespective of the p53 status, thus offering the possibility to target even therapy-resistant or metastatic ES. The combined effectiveness of AUY-VE originated from (i) a depletion of DDR proteins and an accumulation of DNA damages, (ii) pro-apoptotic gene transcription and mitochondrial apoptosis in p53^wt^ cells, but high level of pro-apoptotic ER stress and autophagy in p53^−/−^ cells, and (iii) globally impaired PI3K/AKT/mTOR signaling. BALB-CTA tests and experiments in colon cancer cells showed the therapeutic potential of AUY-VE beyond ES cells. Since HSP90i and ATRi are already used to treat various types of cancer in clinical phase I/II trials [13, 14, 63], their combinations could further increase their effectiveness and improve the prognosis of patients, especially for BRCA- and p53-deficient tumor entities.

## Supplementary Information

**Additional file 1: Figure S1:** Analysis of apoptosis and DDR signaling in ES cells. (A) Analysis p53 expression level by Western blot. β-actin was used to control protein loading. p53 null SaOS-2 cells were treated with the indicated concentrations of AUY922, VE821, KU55933 and their combinations. DMSO was used as control. (A-C) The loss of the mitochondrial transmembrane potential (ΔΨ_M_) and cell death were assessed by flow cytometry after 48 h. All graphs show the mean ± SEM of three independent experiments. Statistical analysis was done using two-way ANOVA tests with GraphPad (*p < 0.05; **p < 0.01; ***p < 0.001). WE-68 (D) and A673 (E) cells were treated with 30 nM of AUY922 alone and in combination with 1–5 µM of VE821 or 2–10 µM of KU55933. Analysis of indicated proteins was done by Western blot after 24 h; α-tubulin and β-actin were used to control protein loading. Immunoblots are representative of at least two independent experiments.

**Additional file 2: Figure S2:** Analysis of cell proliferation. (A) WE-68 and (B) A673 cells were treated with 15–45 nM of AUY922, 5 µM of VE821 and their combinations for up to 72 h. Cell densities were analyzed and quantified using crystal violet staining. A-B show the mean ± SEM of three independent experiments (*p < 0.05; **p < 0.01; ***p < 0.001).

**Additional file 3: Figure S3:** Analysis of cell cycle distribution. (A) WE-68 and (B) A673 cells were treated with 15–45 nM of AUY922, 2 µM of VE821, 5 µM of KU55933 and their combinations for 24 h. (C-E) WE-68 and A673 cells were treated with indicated concentrations of AUY922 (C), VE821 (D) or KU55933 (E) for 48 h. Cell cycle distributions were determined by flow-cytometric analysis of PI-stained ethanol-fixed cells. All graphs show the mean ± SEM of at least two independent experiments.

**Additional file 4: Figure S4.** Analysis of molecular alterations. (A) Otherwise isogenic p53 wild-type (wt) and p53 null (p53-/-) HCT116 cells were treated with 45 nM AUY922 ± 2 µM VE821 for 24 h. Analysis of indicated proteins was done by Western blot. ⍺-tubulin and vinculin were used to control protein loading. Immunoblots are representative for at least two independent experiments. (B) Schematic workflow of the BALB/c cell transformation assay (BALB-CTA). WE-68 cells were treated with 30 nM of AUY922, 1 µM of VE821, 7.5 µM of KU55933 and their combinations; A673 cells were treated with 15 nM of AUY922, 1 µM of VE821, 5 µM of KU55933 and their combinations. DMSO was used for control. (C-D) The mRNA expression of indicated gene was analyzed after 24 h by qPCR. All graphs show the mean ± SEM of three independent experiments (* p < 0.05; ** p < 0.01; *** p < 0.001).

**Additional file 5: Figure S5.** Analysis of ER stress. WE-68 and A673 cells were treated with 15–45 nM of AUY922, 2 µM of VE821, 5 µM of KU55933 and their combinations for 24 h. (A) Intracellular reactive oxygen species (ROS) level were analyzed by flow cytometry using CM-H_2_DCFDA. Both graphs show the mean ± SEM of three independent experiments. (B) Quantification of LAMP1 analyzed by Western blot (see Fig. 4) is shown representing the mean ± SEM of two independent experiments. (C) Otherwise isogenic p53 wild-type (wt) and p53 null (p53-/-) HCT116 cells were treated with 45 nM AUY922 ± 2 µM VE821 for 24 h. Analysis of indicated proteins was done by Western blot. ⍺-tubulin and vinculin were used to control protein loading. Immunoblots are representative for at least two independent experiments.

**Additional file 6: Figure S6.** Accumulation of intracellular defects in WE-68 cells. WE-68 cells were treated with 45 nM AUY922 (B), 2 µM VE821 (C) and their combination (D). DMSO was used for control (A). Intracellular structures were analyzed by transmission electron microscopy (TEM) and are labeled in red: lysosome (lyso), mitochondria (mito), endoplasmic reticulum (ER), nucleus (nuc), autophagosomes (auto), β-glycagon granule (glycagon), lipid droplets, vesicles and lipid-filled vesicles. Red arrows indicate sites of cell rupture. TEM pictures show one representative experiment.

**Additional file 7: Figure S7.** Accumulation of intracellular defects in A673 cells. A673 cells were treated with 45 nM AUY922 (B), 2 µM VE821 (C) and their combination (D). DMSO was used for control (A). Intracellular structures were analyzed by transmission electron microscopy (TEM) and are labeled in red: lysosome (lyso), mitochondria (mito), endoplasmic reticulum (ER), nucleus (nuc), autophagosomes (auto), β-glycagon granule (glycagon), lipid droplets, vesicles and lipid-filled vesicles. A673 cells were treated with 0.4–5 µg/ml of tunicamycin for 24 h. (E) Analysis of indicated proteins was done by Western blot. α-tubulin was used to control protein loading. TEM pictures show one representative experiment; immunoblots are representative for at least two independent experiments.

**Additional file 8: Figure S8.** Proteome analysis in A673 cells. A673 cells were treated with 45 nM AUY922 ± 2 µM VE821 for 24 h, and a quantitative whole proteome analysis was done by mass spectrometry from three individual experiments. Volcano plots show highly altered proteins (cutoff: q < 0.05; log2-fold change > 0.5) after AUY922 (A), VE821 (B) and their combination (AUY922 + VE821) (C). (D) A principal component analysis (PCA) was done on the proteome data set using R studio. The three replicates of each treatment are shown together with their median value (biggest symbol): DMSO (control) in blue, AUY922 in pink, VE821 in green and AUY922 + VE821 in magenta. The circles around each group were inserted by hand after PCA for better visual separation.

**Additional file 9: Figure S9.** Impaired PI3K/AKT/mTOR signaling after AUY-VE. WE-68 (A) and otherwise isogenic p53 wild-type (wt) and p53 null (p53-/-) HCT116 (B) cells were treated with 45 nM AUY922 ± 2 µM VE821. Analysis of indicated proteins was done by Western blot after 24 h. Vinculin was used to control protein loading. Immunoblots are representative for at least two independent experiments.

**Additional file 10: Figure S10.** Ingenuity pathway analysis of the proteome data set. A673 cells were treated with 45 nM AUY922 ± 2 µM VE821 for 24 h, and a quantitative whole proteome analysis was done by mass spectrometry from three individual experiments. The proteome data set was further processed by ingenuity pathway analysis (IPA; cutoff: q < 0.05) for toxicities after AUY922 (A) or VE821 (B) treatments. The common set of 208 overlapping differentially expressed proteins (DEPs) between AUY922-VE821 combinations (AUY-VE) and single treatments was processed by IPA for diseases and biofunctions (C) and toxicities (D). The unique set of 630 DEPs after AUY-VE treatments was processed by IPA for diseases and biofunctions (E) and toxicities (F).

**Additional file 11: File S1**. Proteome alterations in A673 cells induced by AUY922, VE821 and AUY-VE.

## Data Availability

The mass spectrometry proteomics data have been deposited to the ProteomeXchange Consortium (http://proteomecentral.proteomexchange.org) [67–69] via the PRIDE partner repository [70] with the data set identifier PXD021562. Otherwise, all data generated or analyzed during this study are included in this published article (and its supporting information files).

## Abbreviations

ATMi: ATM inhibitor
ATRi: ATR inhibitor
AUY-KU: Combined treatment with AUY922 and KU55933
AUY-VE: Combined treatment with AUY922 and VE821
BALB-CTA: BALB/c cell transformation assay
cl: Cleaved
CMA: Chaperone-mediated autophagy
DDR: DNA damage response
DEPs: Differentially expressed proteins
ER: Endoplasmic reticulum
ES: Ewing's sarcoma
FA: Fatty acids
HR: Homologous recombination
HSP90i: HSP90 inhibitor
IPA: Ingenuity pathway analysis
p53^−^^/^^−^: P53 null
p53^wt^: P53 wild-type
ROS: Reactive oxygen species
RS: Replicative stress
SSBs: Single strand breaks
UPR: Unfolded protein response
