# Supplementary material for: Cooperative treatment effectiveness of ATR and HSP90 inhibition in Ewing’s sarcoma cells
Source: Cell Biosci. 2021 Mar 20;11:57. doi: 10.1186/s13578-021-00571-y (PMC7981928; doi:10.1186/s13578-021-00571-y)
Supplement: Supplementary file 1 — Additional file 1: Figure S1: Analysis of apoptosis and DDR signaling in ES cells. (A) Analysis p53 expression level by Western blot. β-actin was used to control protein loading. p53 null SaOS-2 cells were treated with the indicated concentrations of AUY922, VE821, KU55933 and their combinations. DMSO was used as control. (A-C) The loss of the mitochondrial transmembrane potential (ΔΨM) and cell death were assessed by flow cytometry after 48 h. All graphs show the mean ± SEM of three independent experiments. Statistical analysis was done using two-way ANOVA tests with GraphPad (*p < 0.05; **p < 0.01; ***p < 0.001). WE-68 (D) and A673 (E) cells were treated with 30 nM of AUY922 alone and in combination with 1–5 µM of VE821 or 2–10 µM of KU55933. Analysis of indicated proteins was done by Western blot after 24 h; α-tubulin and β-actin were used to control protein loading. Immunoblots are representative of at least two independent experiments. [file 13578_2021_571_MOESM1_ESM.pptx]

## Slide 1
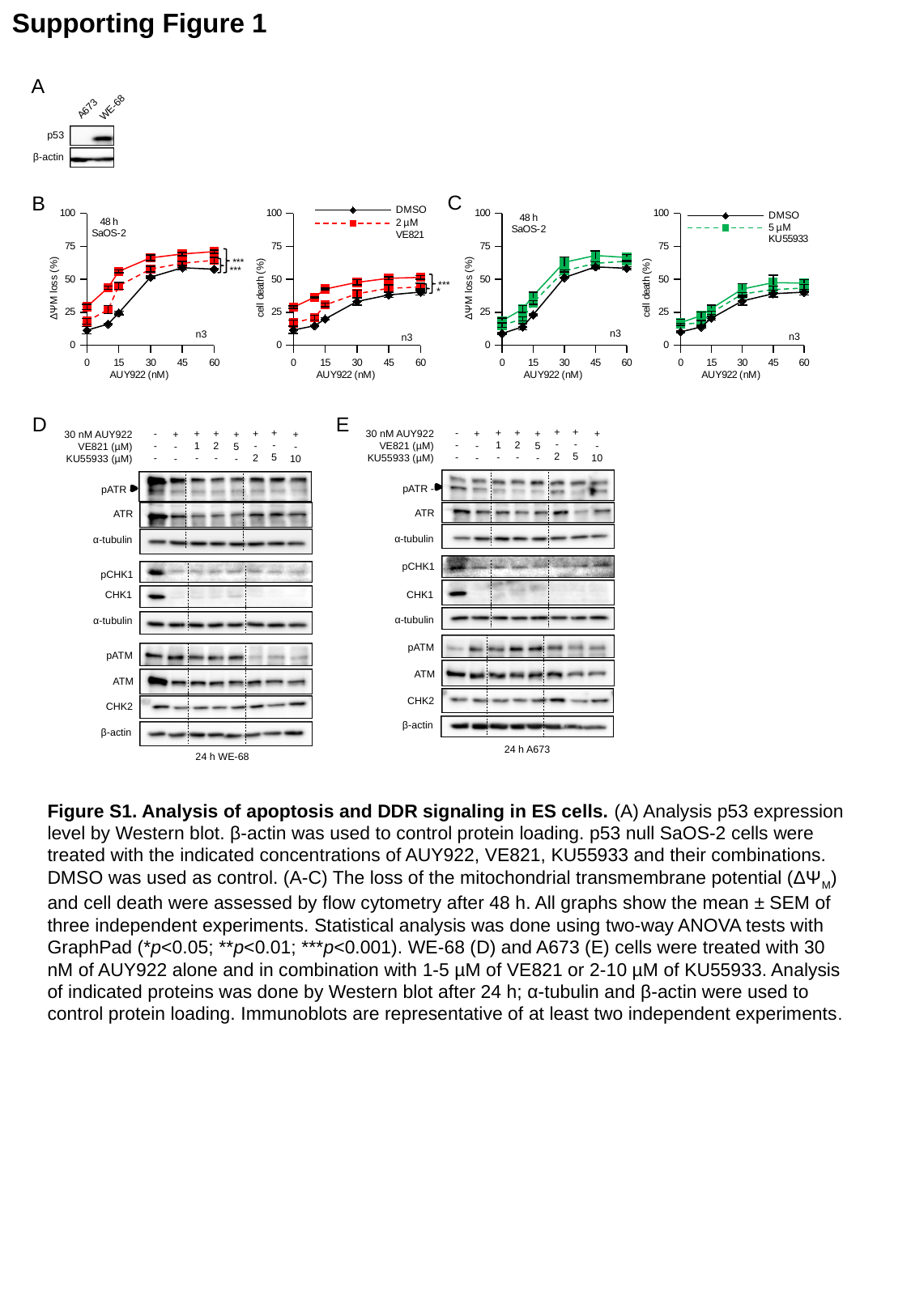

Supporting Figure 1
A
WE-68
A673
p53
β-actin
C
B
### Chart
| Category | | | |
|---|---|---|---|
### Chart
| Category | | | |
|---|---|---|---|
### Chart
| Category | | | |
|---|---|---|---|
### Chart
| Category | | | |
|---|---|---|---|
***
***
***
*
n3
n3
n3
n3
E
D
+
-
5
+
-
2
+
2
-
-
-
-
+
1
-
+
5
-
30 nM AUY922
VE821 (µM)
KU55933 (µM)
+
-
-
+
-
10
pATR -
ATR
α-tubulin
pCHK1
CHK1
α-tubulin
pATM
ATM
CHK2
β-actin
24 h A673
+
-
5
+
-
2
+
2
-
-
-
-
+
1
-
+
5
-
30 nM AUY922
VE821 (µM)
KU55933 (µM)
+
-
-
+
-
10
pATR -
ATR
α-tubulin
pCHK1
CHK1
α-tubulin
pATM
ATM
CHK2
β-actin
24 h WE-68
Figure S1. Analysis of apoptosis and DDR signaling in ES cells. (A) Analysis p53 expression level by Western blot. β-actin was used to control protein loading. p53 null SaOS-2 cells were treated with the indicated concentrations of AUY922, VE821, KU55933 and their combinations. DMSO was used as control. (A-C) The loss of the mitochondrial transmembrane potential (ΔΨM) and cell death were assessed by flow cytometry after 48 h. All graphs show the mean ± SEM of three independent experiments. Statistical analysis was done using two-way ANOVA tests with GraphPad (*p<0.05; **p<0.01; ***p<0.001). WE-68 (D) and A673 (E) cells were treated with 30 nM of AUY922 alone and in combination with 1-5 µM of VE821 or 2-10 µM of KU55933. Analysis of indicated proteins was done by Western blot after 24 h; α-tubulin and β-actin were used to control protein loading. Immunoblots are representative of at least two independent experiments.
