# Supplementary material for: Cooperative treatment effectiveness of ATR and HSP90 inhibition in Ewing’s sarcoma cells
Source: Cell Biosci. 2021 Mar 20;11:57. doi: 10.1186/s13578-021-00571-y (PMC7981928; doi:10.1186/s13578-021-00571-y)
Supplement: Supplementary file 2 — Additional file 2: Figure S2: Analysis of cell proliferation. (A) WE-68 and (B) A673 cells were treated with 15–45 nM of AUY922, 5 µM of VE821 and their combinations for up to 72 h. Cell densities were analyzed and quantified using crystal violet staining. A-B show the mean ± SEM of three independent experiments (*p < 0.05; **p < 0.01; ***p < 0.001). [file 13578_2021_571_MOESM2_ESM.pptx]

## Slide 1
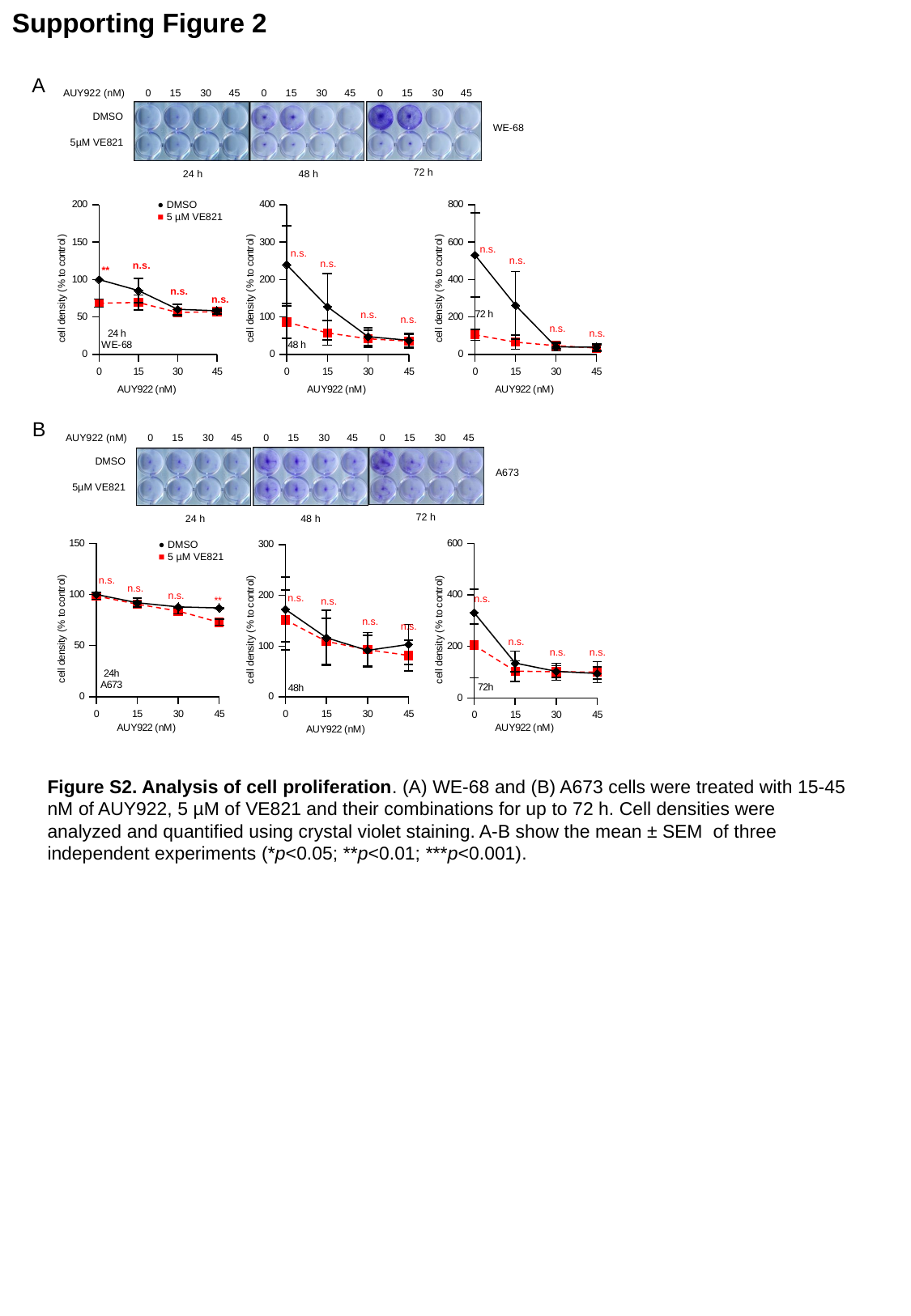

Supporting Figure 2
A
AUY922 (nM)
15
30
45
15
30
45
15
30
45
0
0
0
DMSO
WE-68
5µM VE821
72 h
24 h
48 h
### Chart: 48 h
| Category | | |
|---|---|---|n.s.
n.s.
n.s.
n.s.
### Chart: 72 h
| Category | | |
|---|---|---|n.s.
n.s.
n.s.
n.s.
### Chart: 24 h
WE-68
| Category | | |
|---|---|---|n.s.
**
n.s.
n.s.
 ● DMSO
 ■ 5 µM VE821
B
AUY922 (nM)
15
30
45
15
30
45
15
30
45
0
0
0
DMSO
A673
5µM VE821
72 h
24 h
48 h
### Chart: 24h
A673
| Category | | |
|---|---|---|n.s.
n.s.
n.s.
**
### Chart: 72h
| Category | | |
|---|---|---|n.s.
n.s.
n.s.
n.s.
 ● DMSO
 ■ 5 µM VE821
### Chart: 48h
| Category | | |
|---|---|---|n.s.
n.s.
n.s.
n.s.
Figure S2. Analysis of cell proliferation. (A) WE-68 and (B) A673 cells were treated with 15-45 nM of AUY922, 5 µM of VE821 and their combinations for up to 72 h. Cell densities were analyzed and quantified using crystal violet staining. A-B show the mean ± SEM of three independent experiments (*p<0.05; **p<0.01; ***p<0.001).
