# Supplementary material for: Cooperative treatment effectiveness of ATR and HSP90 inhibition in Ewing’s sarcoma cells
Source: Cell Biosci. 2021 Mar 20;11:57. doi: 10.1186/s13578-021-00571-y (PMC7981928; doi:10.1186/s13578-021-00571-y)
Supplement: Supplementary file 3 — Additional file 3: Figure S3: Analysis of cell cycle distribution. (A) WE-68 and (B) A673 cells were treated with 15–45 nM of AUY922, 2 µM of VE821, 5 µM of KU55933 and their combinations for 24 h. (C-E) WE-68 and A673 cells were treated with indicated concentrations of AUY922 (C), VE821 (D) or KU55933 (E) for 48 h. Cell cycle distributions were determined by flow-cytometric analysis of PI-stained ethanol-fixed cells. All graphs show the mean ± SEM of at least two independent experiments. [file 13578_2021_571_MOESM3_ESM.pptx]

## Slide 1
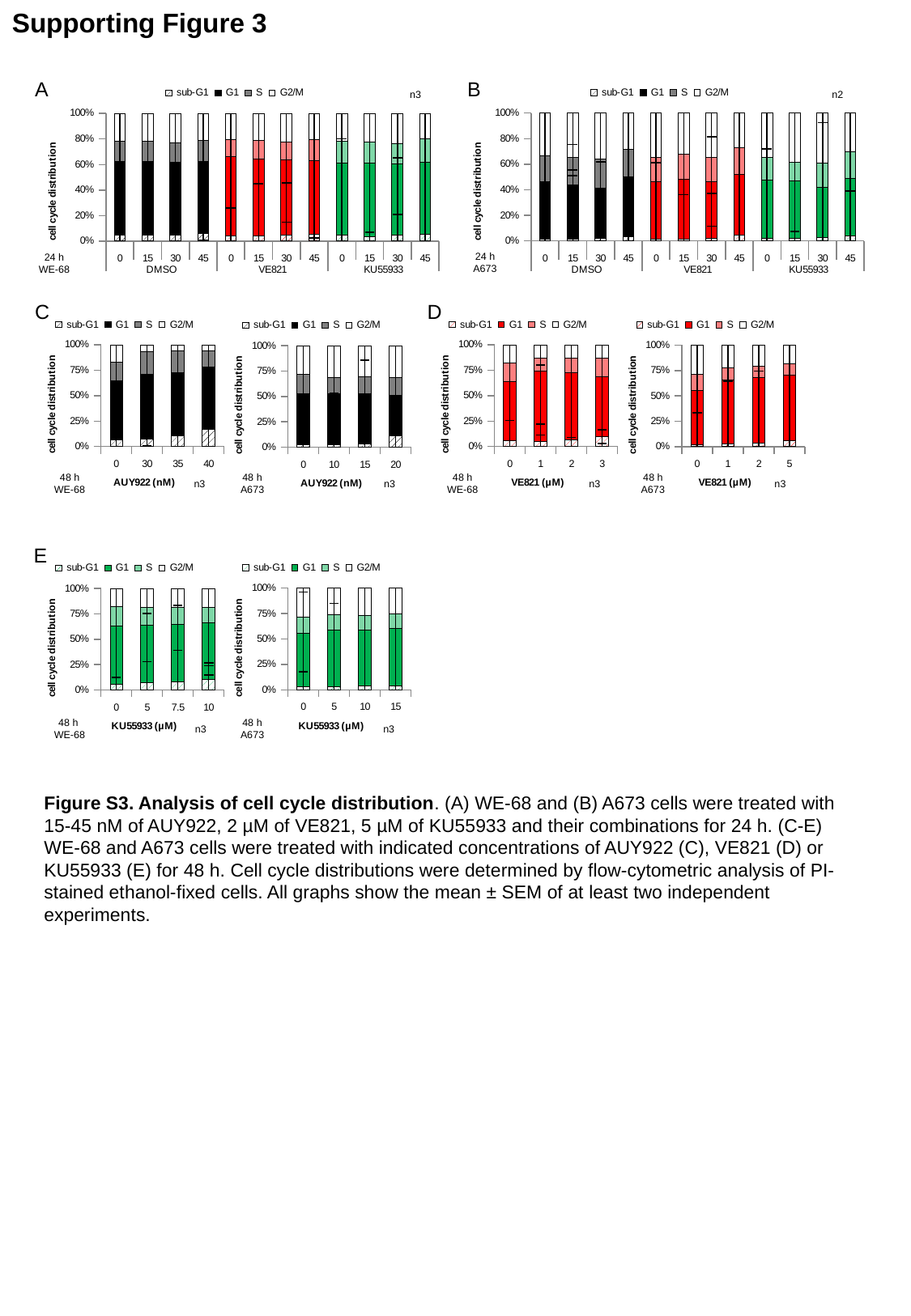

Supporting Figure 3
A
B
### Chart
| Category | | | | |
|---|---|---|---|---|
| 0 | 1.5 | 44.9 | 20.15 | 33.4 |
| 15 | 1.1 | 42.849999999999994 | 21.299999999999997 | 34.45 |
| 30 | 2.0 | 39.3 | 22.95 | 35.85 |
| 45 | 3.0 | 46.95 | 21.9 | 28.5 |
| 0 | 1.2999999999999998 | 45.15 | 18.85 | 34.65 |
| 15 | 1.25 | 46.599999999999994 | 19.799999999999997 | 32.25 |
| 30 | 2.15 | 44.45 | 19.15 | 34.5 |
| 45 | 4.45 | 47.35 | 21.35 | 26.950000000000003 |
| 0 | 1.8 | 44.65 | 17.45 | 34.3 |
| 15 | 2.25 | 44.95 | 14.450000000000001 | 38.3 |
| 30 | 2.5 | 39.45 | 19.35 | 38.85 |
| 45 | 3.95 | 44.849999999999994 | 21.2 | 30.0 |
### Chart
| Category | | | | |
|---|---|---|---|---|
| 0 | 4.466666666666666 | 57.43333333333334 | 15.666666666666666 | 21.46666666666667 |
| 15 | 4.7 | 57.23333333333333 | 15.4 | 21.599999999999998 |
| 30 | 4.566666666666667 | 56.53333333333334 | 14.799999999999999 | 23.0 |
| 45 | 6.033333333333334 | 55.53333333333333 | 16.433333333333334 | 20.833333333333332 |
| 0 | 4.066666666666667 | 61.86666666666667 | 12.933333333333332 | 20.466666666666665 |
| 15 | 4.233333333333333 | 59.93333333333334 | 14.1 | 21.099999999999998 |
| 30 | 4.633333333333334 | 58.53333333333333 | 13.933333333333332 | 21.900000000000002 |
| 45 | 5.1 | 57.46666666666667 | 15.933333333333335 | 20.3 |
| 0 | 4.3999999999999995 | 55.93333333333334 | 16.666666666666668 | 21.7 |
| 15 | 3.733333333333333 | 57.0 | 16.533333333333335 | 21.933333333333334 |
| 30 | 4.3999999999999995 | 55.29999999999999 | 15.933333333333332 | 23.266666666666666 |
| 45 | 5.1000000000000005 | 55.666666666666664 | 17.933333333333334 | 19.833333333333332 |n3
n2
24 h
A673
24 h
WE-68
C
D
### Chart
| Category | | | | |
|---|---|---|---|---|
| | 6.5 | 58.400000000000006 | 18.133333333333336 | 16.933333333333334 |
| | 6.800000000000001 | 56.9 | 19.833333333333332 | 6.1625 |
| | 9.299999999999999 | 56.199999999999996 | 19.400000000000002 | 5.675 |
| | 15.100000000000001 | 56.26666666666666 | 14.366666666666667 | 5.35 |
### Chart
| Category | | | | |
|---|---|---|---|---|
| 0 | 5.5 | 58.26666666666667 | 18.6 | 17.633333333333333 |
| 1 | 4.566666666666666 | 69.36666666666667 | 12.733333333333334 | 13.333333333333334 |
| 2 | 6.833333333333333 | 66.06666666666666 | 14.1 | 13.033333333333331 |
| 3 | 9.7 | 59.0 | 18.2 | 13.133333333333333 |
### Chart
| Category | | | | |
|---|---|---|---|---|
| 0 | 2.2333333333333334 | 53.26666666666667 | 15.933333333333332 | 28.566666666666666 |
| 1 | 2.3666666666666667 | 62.9 | 12.4 | 22.333333333333332 |
| 2 | 3.3333333333333335 | 64.39999999999999 | 11.6 | 20.7 |
| 5 | 5.633333333333333 | 64.63333333333333 | 10.966666666666669 | 18.766666666666666 |
### Chart
| Category | | | | |
|---|---|---|---|---|
| 0 | 2.3333333333333335 | 50.4 | 18.8 | 28.433333333333334 |
| 10 | 2.3333333333333335 | 50.26666666666667 | 16.266666666666666 | 31.133333333333336 |
| 15 | 3.5666666666666664 | 49.03333333333334 | 16.433333333333334 | 30.900000000000002 |
| 20 | 10.899999999999999 | 40.46666666666666 | 17.233333333333334 | 31.433333333333334 |48 h
A673
48 h
WE-68
48 h
WE-68
48 h
A673
n3
n3
n3
n3
E
### Chart
| Category | | | | |
|---|---|---|---|---|
| 0 | 2.8000000000000003 | 53.133333333333326 | 15.533333333333333 | 28.53333333333333 |
| 5 | 2.966666666666667 | 55.666666666666664 | 15.133333333333333 | 26.3 |
| 10 | 3.4666666666666663 | 55.300000000000004 | 14.633333333333333 | 26.633333333333336 |
| 15 | 4.066666666666666 | 56.199999999999996 | 14.200000000000001 | 25.5 |
### Chart
| Category | | | | |
|---|---|---|---|---|
| 0 | 5.8999999999999995 | 57.26666666666667 | 18.76666666666667 | 18.066666666666666 |
| 5 | 7.033333333333332 | 57.03333333333333 | 17.3 | 18.633333333333333 |
| 7.5 | 7.866666666666667 | 56.76666666666667 | 16.933333333333334 | 18.46666666666667 |
| 10 | 10.466666666666667 | 55.76666666666667 | 15.5 | 18.3 |48 h
WE-68
48 h
A673
n3
n3
Figure S3. Analysis of cell cycle distribution. (A) WE-68 and (B) A673 cells were treated with 15-45 nM of AUY922, 2 µM of VE821, 5 µM of KU55933 and their combinations for 24 h. (C-E) WE-68 and A673 cells were treated with indicated concentrations of AUY922 (C), VE821 (D) or KU55933 (E) for 48 h. Cell cycle distributions were determined by flow-cytometric analysis of PI-stained ethanol-fixed cells. All graphs show the mean ± SEM of at least two independent experiments.
