# Supplementary material for: Cooperative treatment effectiveness of ATR and HSP90 inhibition in Ewing’s sarcoma cells
Source: Cell Biosci. 2021 Mar 20;11:57. doi: 10.1186/s13578-021-00571-y (PMC7981928; doi:10.1186/s13578-021-00571-y)
Supplement: Supplementary file 4 — Additional file 4: Figure S4. Analysis of molecular alterations. (A) Otherwise isogenic p53 wild-type (wt) and p53 null (p53-/-) HCT116 cells were treated with 45 nM AUY922 ± 2 µM VE821 for 24 h. Analysis of indicated proteins was done by Western blot. ⍺-tubulin and vinculin were used to control protein loading. Immunoblots are representative for at least two independent experiments. (B) Schematic workflow of the BALB/c cell transformation assay (BALB-CTA). WE-68 cells were treated with 30 nM of AUY922, 1 µM of VE821, 7.5 µM of KU55933 and their combinations; A673 cells were treated with 15 nM of AUY922, 1 µM of VE821, 5 µM of KU55933 and their combinations. DMSO was used for control. (C-D) The mRNA expression of indicated gene was analyzed after 24 h by qPCR. All graphs show the mean ± SEM of three independent experiments (* p < 0.05; ** p < 0.01; *** p < 0.001). [file 13578_2021_571_MOESM4_ESM.pptx]

## Slide 1
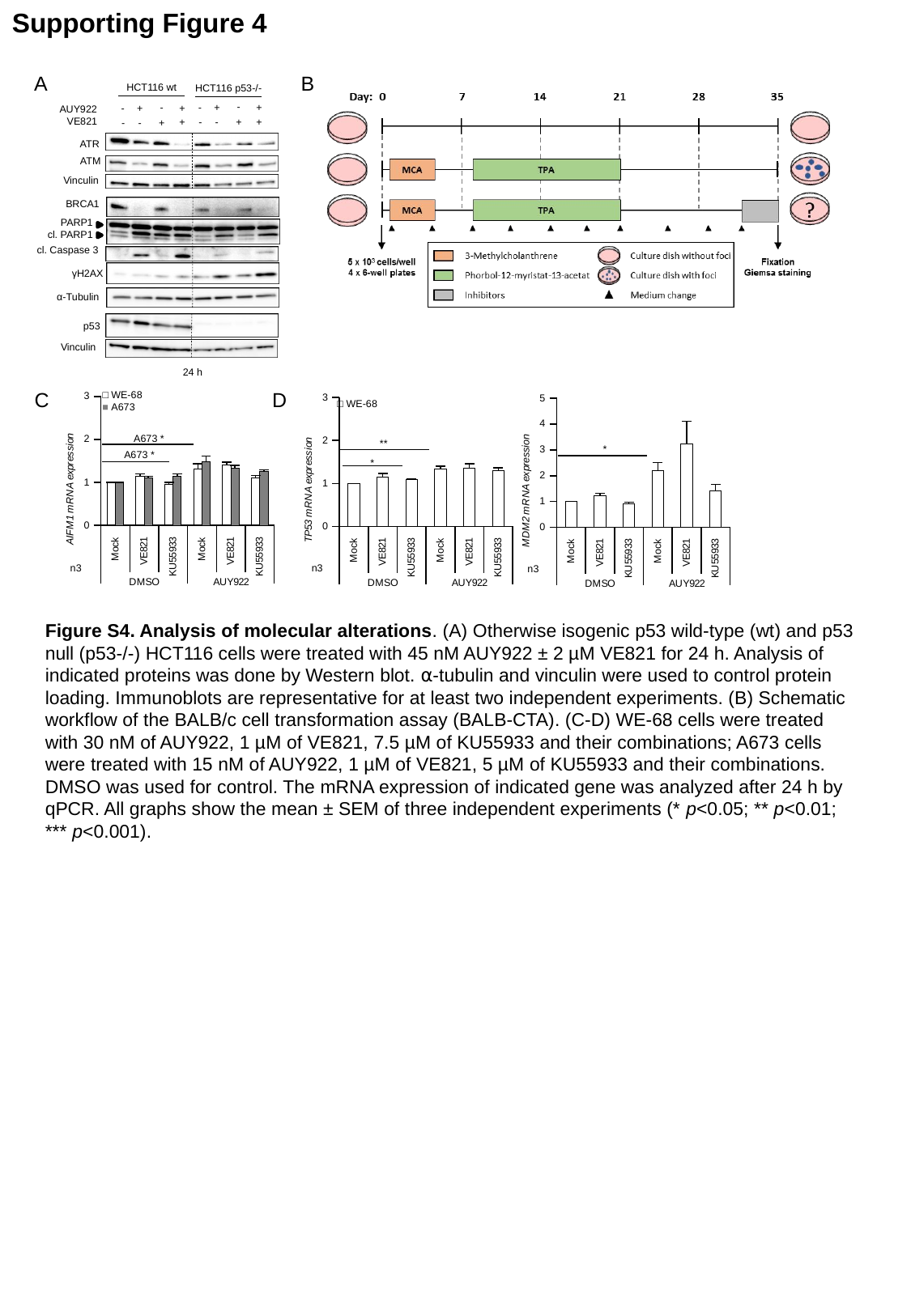

Supporting Figure 4
A
B
HCT116 wt
HCT116 p53-/-
-
-
+
-
+
+
-
+
AUY922
VE821
+
-
-
+
+
-
-
+
ATR
ATM
Vinculin
BRCA1
PARP1
cl. PARP1
cl. Caspase 3
γH2AX
α-Tubulin
p53
Vinculin
24 h
C
D
 □ WE-68
 ■ A673
### Chart
| Category | | |
|---|---|---|
| Mock | 1.0 | 1.0 |
| VE821 | 1.1470546038166312 | 1.0986899611177403 |
| KU55933 | 0.9492737379227089 | 1.1519865399972387 |
| Mock | 1.3178214423401744 | 1.4754648244374504 |
| VE821 | 1.4053721951595215 | 1.3329816469821794 |
| KU55933 | 1.1019952450080754 | 1.2483032455231269 |A673 *
A673 *
### Chart
| Category | |
|---|---|
| Mock | 1.0 |
| VE821 | 1.1387309961545076 |
| KU55933 | 1.084433634064643 |
| Mock | 1.341347029449883 |
| VE821 | 1.344667920541145 |
| KU55933 | 1.3035003312795193 |**
*
### Chart
| Category | |
|---|---|
| Mock | 1.0 |
| VE821 | 1.2098997214638774 |
| KU55933 | 0.9085670180370805 |
| Mock | 2.184207320848126 |
| VE821 | 3.2385766766914017 |
| KU55933 | 1.4251108864003852 |*
 □ WE-68
n3
n3
n3
Figure S4. Analysis of molecular alterations. (A) Otherwise isogenic p53 wild-type (wt) and p53 null (p53-/-) HCT116 cells were treated with 45 nM AUY922 ± 2 µM VE821 for 24 h. Analysis of indicated proteins was done by Western blot. ⍺-tubulin and vinculin were used to control protein loading. Immunoblots are representative for at least two independent experiments. (B) Schematic workflow of the BALB/c cell transformation assay (BALB-CTA). (C-D) WE-68 cells were treated with 30 nM of AUY922, 1 µM of VE821, 7.5 µM of KU55933 and their combinations; A673 cells were treated with 15 nM of AUY922, 1 µM of VE821, 5 µM of KU55933 and their combinations. DMSO was used for control. The mRNA expression of indicated gene was analyzed after 24 h by qPCR. All graphs show the mean ± SEM of three independent experiments (* p<0.05; ** p<0.01; *** p<0.001).
