# Supplementary material for: Cooperative treatment effectiveness of ATR and HSP90 inhibition in Ewing’s sarcoma cells
Source: Cell Biosci. 2021 Mar 20;11:57. doi: 10.1186/s13578-021-00571-y (PMC7981928; doi:10.1186/s13578-021-00571-y)
Supplement: Supplementary file 5 — Additional file 5: Figure S5. Analysis of ER stress. WE-68 and A673 cells were treated with 15–45 nM of AUY922, 2 µM of VE821, 5 µM of KU55933 and their combinations for 24 h. (A) Intracellular reactive oxygen species (ROS) level were analyzed by flow cytometry using CM-H2DCFDA. Both graphs show the mean ± SEM of three independent experiments. (B) Quantification of LAMP1 analyzed by Western blot (see Fig. 4) is shown representing the mean ± SEM of two independent experiments. (C) Otherwise isogenic p53 wild-type (wt) and p53 null (p53-/-) HCT116 cells were treated with 45 nM AUY922 ± 2 µM VE821 for 24 h. Analysis of indicated proteins was done by Western blot. ⍺-tubulin and vinculin were used to control protein loading. Immunoblots are representative for at least two independent experiments. [file 13578_2021_571_MOESM5_ESM.pptx]

## Slide 1
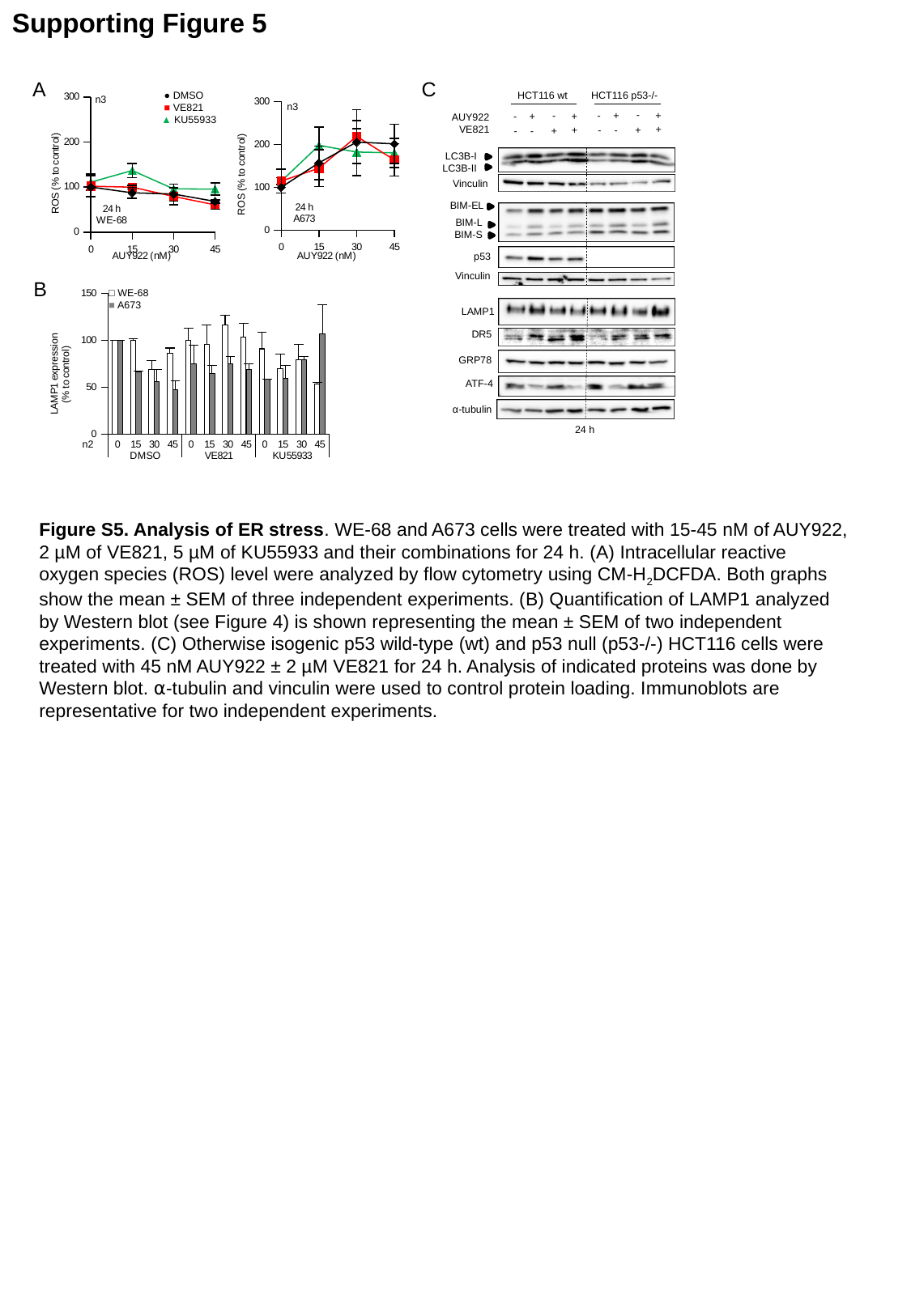

Supporting Figure 5
A
C
 ● DMSO
 ■ VE821
▲ KU55933
HCT116 wt
HCT116 p53-/-
-
-
+
-
+
+
-
+
AUY922
VE821
+
-
-
+
+
-
-
+
LC3B-I
LC3B-II
Vinculin
BIM-EL
BIM-L
BIM-S
p53
Vinculin
LAMP1
DR5
GRP78
ATF-4
α-tubulin
24 h
### Chart
| Category | | | |
|---|---|---|---|
### Chart
| Category | | | |
|---|---|---|---|n3
n3
B
 □ WE-68
 ■ A673
### Chart
| Category | | |
|---|---|---|
| 0 | 100.0 | 100.0 |
| 15 | 99.36177249721655 | 66.62121621847291 |
| 30 | 68.57498807321434 | 56.11307299101844 |
| 45 | 85.87260947025894 | 47.17661838415461 |
| 0 | 99.70307112336526 | 74.96002620688029 |
| 15 | 95.2451733685566 | 64.66391091038629 |
| 30 | 116.18068532907506 | 74.93483799320316 |
| 45 | 103.19254942752653 | 68.42004019869545 |
| 0 | 90.72381899786278 | 58.40560420783984 |
| 15 | 69.35565465838127 | 59.57325623835685 |
| 30 | 79.39610521776166 | 79.23293164673055 |
| 45 | 53.31497605813745 | 106.9250241971082 |n2
Figure S5. Analysis of ER stress. WE-68 and A673 cells were treated with 15-45 nM of AUY922, 2 µM of VE821, 5 µM of KU55933 and their combinations for 24 h. (A) Intracellular reactive oxygen species (ROS) level were analyzed by flow cytometry using CM-H2DCFDA. Both graphs show the mean ± SEM of three independent experiments. (B) Quantification of LAMP1 analyzed by Western blot (see Figure 4) is shown representing the mean ± SEM of two independent experiments. (C) Otherwise isogenic p53 wild-type (wt) and p53 null (p53-/-) HCT116 cells were treated with 45 nM AUY922 ± 2 µM VE821 for 24 h. Analysis of indicated proteins was done by Western blot. ⍺-tubulin and vinculin were used to control protein loading. Immunoblots are representative for two independent experiments.
