# Supplementary material for: Cooperative treatment effectiveness of ATR and HSP90 inhibition in Ewing’s sarcoma cells
Source: Cell Biosci. 2021 Mar 20;11:57. doi: 10.1186/s13578-021-00571-y (PMC7981928; doi:10.1186/s13578-021-00571-y)
Supplement: Supplementary file 6 — Additional file 6: Figure S6. Accumulation of intracellular defects in WE-68 cells. WE-68 cells were treated with 45 nM AUY922 (B), 2 µM VE821 (C) and their combination (D). DMSO was used for control (A). Intracellular structures were analyzed by transmission electron microscopy (TEM) and are labeled in red: lysosome (lyso), mitochondria (mito), endoplasmic reticulum (ER), nucleus (nuc), autophagosomes (auto), β-glycagon granule (glycagon), lipid droplets, vesicles and lipid-filled vesicles. Red arrows indicate sites of cell rupture. TEM pictures show one representative experiment. [file 13578_2021_571_MOESM6_ESM.pptx]

## Slide 1
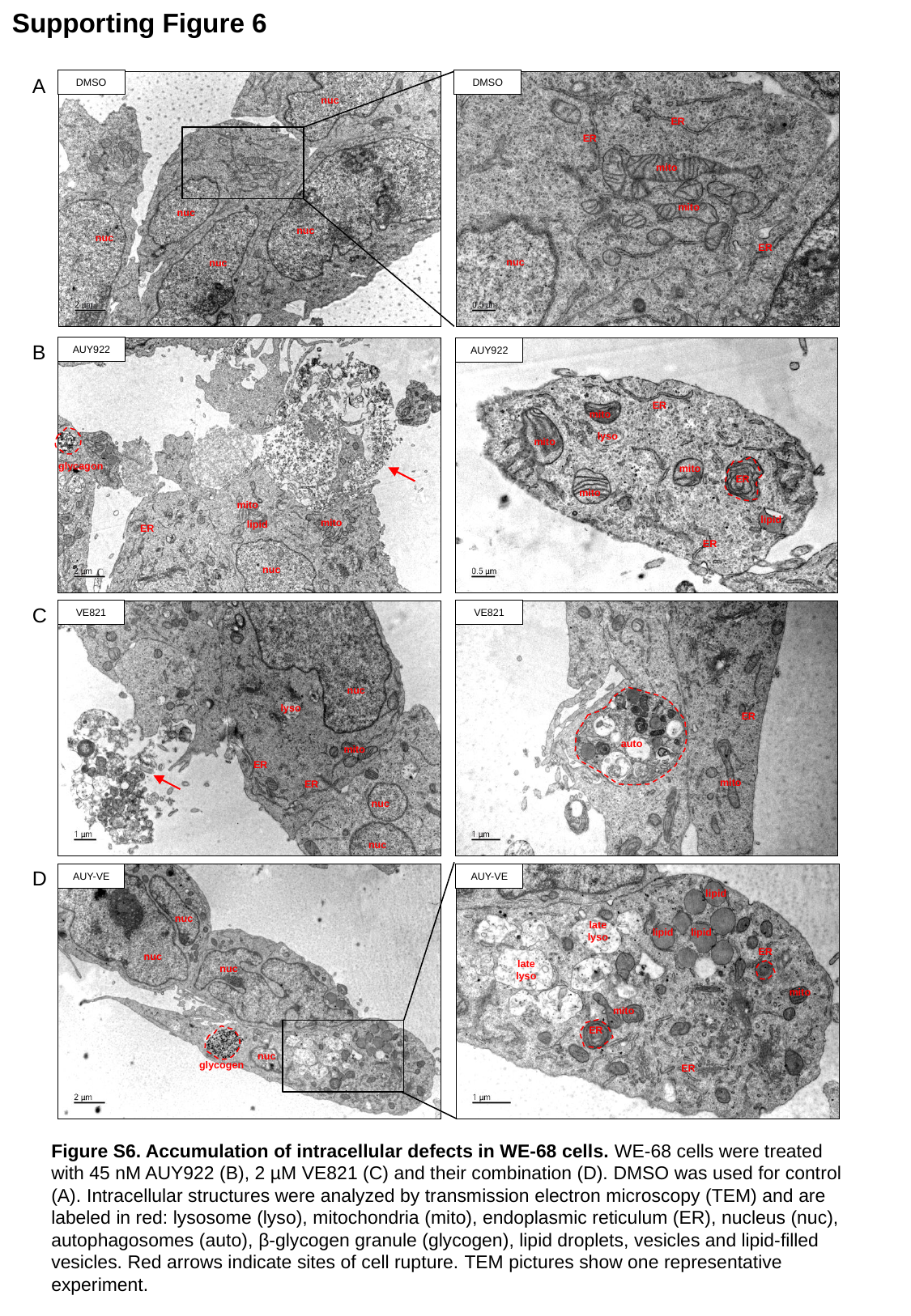

Supporting Figure 6
A
DMSO
DMSO
nuc
ER
ER
mito
mito
nuc
nuc
nuc
ER
nuc
nuc
B
AUY922
AUY922
ER
mito
lyso
mito
glycagon
mito
ER
mito
mito
lipid
mito
lipid
ER
ER
nuc
C
VE821
VE821
nuc
lyso
ER
auto
mito
ER
mito
ER
nuc
nuc
D
AUY-VE
AUY-VE
lipid
nuc
late
lyso
lipid
lipid
ER
nuc
late
lyso
nuc
mito
mito
ER
nuc
glycogen
ER
Figure S6. Accumulation of intracellular defects in WE-68 cells. WE-68 cells were treated with 45 nM AUY922 (B), 2 µM VE821 (C) and their combination (D). DMSO was used for control (A). Intracellular structures were analyzed by transmission electron microscopy (TEM) and are labeled in red: lysosome (lyso), mitochondria (mito), endoplasmic reticulum (ER), nucleus (nuc), autophagosomes (auto), β-glycogen granule (glycogen), lipid droplets, vesicles and lipid-filled vesicles. Red arrows indicate sites of cell rupture. TEM pictures show one representative experiment.
