# Supplementary material for: Cooperative treatment effectiveness of ATR and HSP90 inhibition in Ewing’s sarcoma cells
Source: Cell Biosci. 2021 Mar 20;11:57. doi: 10.1186/s13578-021-00571-y (PMC7981928; doi:10.1186/s13578-021-00571-y)
Supplement: Supplementary file 7 — Additional file 7: Figure S7. Accumulation of intracellular defects in A673 cells. A673 cells were treated with 45 nM AUY922 (B), 2 µM VE821 (C) and their combination (D). DMSO was used for control (A). Intracellular structures were analyzed by transmission electron microscopy (TEM) and are labeled in red: lysosome (lyso), mitochondria (mito), endoplasmic reticulum (ER), nucleus (nuc), autophagosomes (auto), β-glycagon granule (glycagon), lipid droplets, vesicles and lipid-filled vesicles. A673 cells were treated with 0.4–5 µg/ml of tunicamycin for 24 h. (E) Analysis of indicated proteins was done by Western blot. α-tubulin was used to control protein loading. TEM pictures show one representative experiment; immunoblots are representative for at least two independent experiments. [file 13578_2021_571_MOESM7_ESM.pptx]

## Slide 1
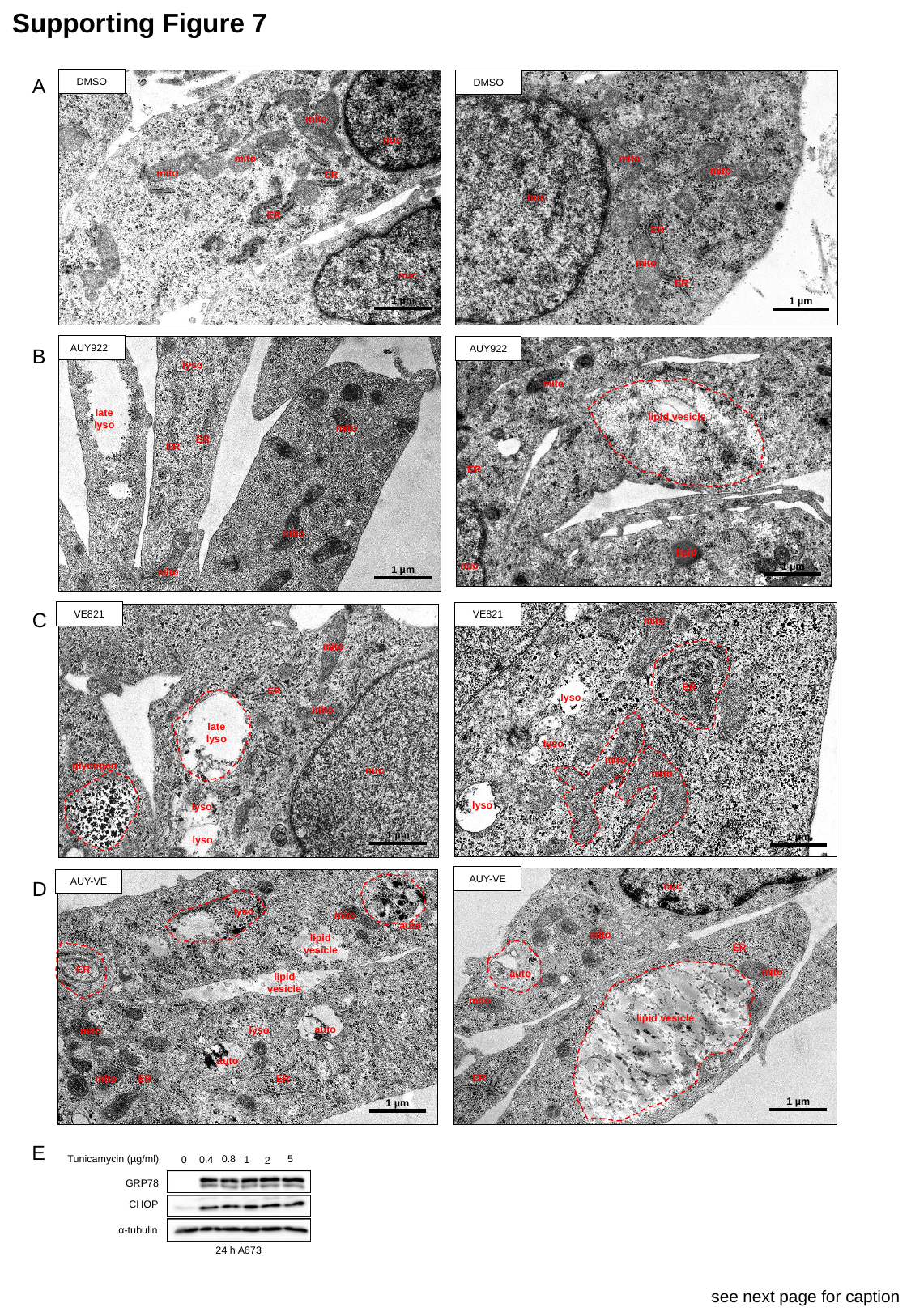

Supporting Figure 7
A
DMSO
mito
nuc
mito
mito
ER
ER
nuc
1 µm
DMSO
mito
mito
nuc
ER
mito
ER
1 µm
AUY922
lyso
late
lyso
mito
ER
ER
mito
1 µm
mito
AUY922
mito
lipid vesicle
ER
lipid
nuc
1 µm
B
C
VE821
mito
ER
mito
late
lyso
glycogen
nuc
lyso
1 µm
lyso
VE821
mito
ER
lyso
lyso
mito
mito
lyso
1 µm
AUY-VE
nuc
mito
ER
mito
auto
mito
lipid vesicle
ER
1 µm
AUY-VE
lyso
mito
auto
lipid
vesicle
ER
lipid
vesicle
auto
lyso
mito
auto
mito
ER
ER
1 µm
D
E
5
Tunicamycin (µg/ml)
0.8
0
0.4
1
2
GRP78
CHOP
α-tubulin
24 h A673
see next page for caption

## Slide 2
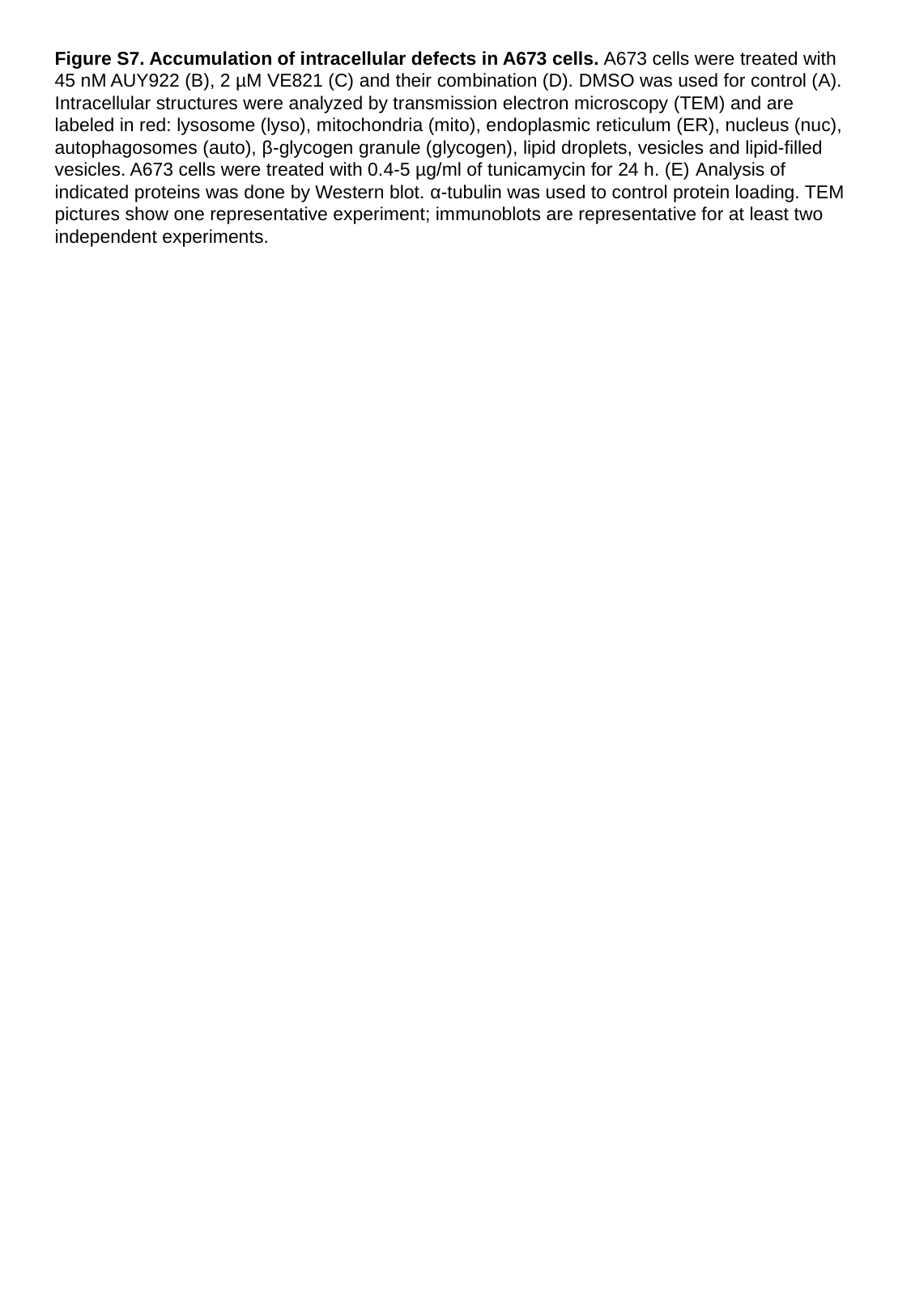

Figure S7. Accumulation of intracellular defects in A673 cells. A673 cells were treated with 45 nM AUY922 (B), 2 µM VE821 (C) and their combination (D). DMSO was used for control (A). Intracellular structures were analyzed by transmission electron microscopy (TEM) and are labeled in red: lysosome (lyso), mitochondria (mito), endoplasmic reticulum (ER), nucleus (nuc), autophagosomes (auto), β-glycogen granule (glycogen), lipid droplets, vesicles and lipid-filled vesicles. A673 cells were treated with 0.4-5 µg/ml of tunicamycin for 24 h. (E) Analysis of indicated proteins was done by Western blot. α-tubulin was used to control protein loading. TEM pictures show one representative experiment; immunoblots are representative for at least two independent experiments.
