# Supplementary material for: Cooperative treatment effectiveness of ATR and HSP90 inhibition in Ewing’s sarcoma cells
Source: Cell Biosci. 2021 Mar 20;11:57. doi: 10.1186/s13578-021-00571-y (PMC7981928; doi:10.1186/s13578-021-00571-y)
Supplement: Supplementary file 8 — Additional file 8: Figure S8. Proteome analysis in A673 cells. A673 cells were treated with 45 nM AUY922 ± 2 µM VE821 for 24 h, and a quantitative whole proteome analysis was done by mass spectrometry from three individual experiments. Volcano plots show highly altered proteins (cutoff: q < 0.05; log2-fold change > 0.5) after AUY922 (A), VE821 (B) and their combination (AUY922 + VE821) (C). (D) A principal component analysis (PCA) was done on the proteome data set using R studio. The three replicates of each treatment are shown together with their median value (biggest symbol): DMSO (control) in blue, AUY922 in pink, VE821 in green and AUY922 + VE821 in magenta. The circles around each group were inserted by hand after PCA for better visual separation. [file 13578_2021_571_MOESM8_ESM.pptx]

## Slide 1
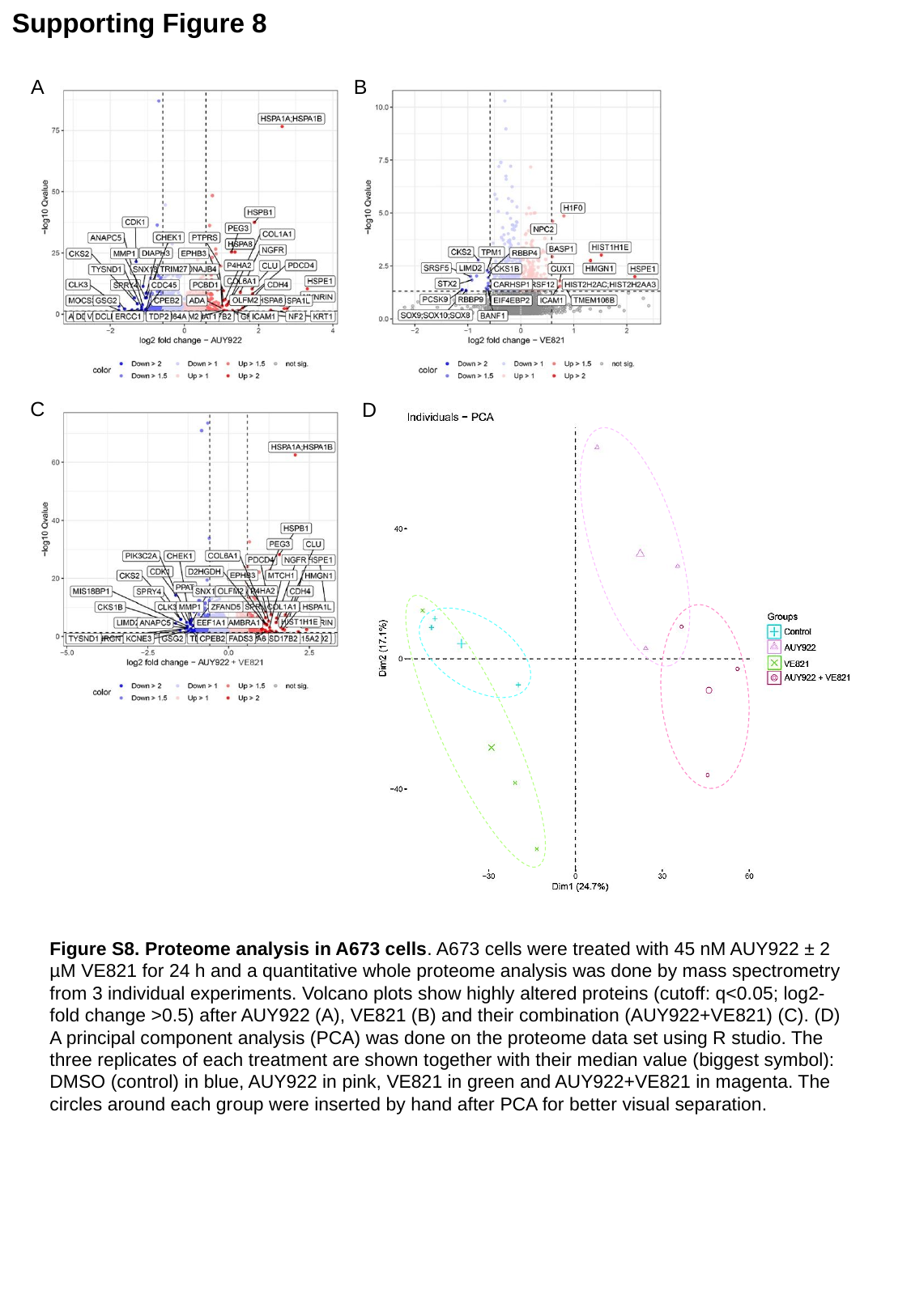

Supporting Figure 8
A
B
C
D
Figure S8. Proteome analysis in A673 cells. A673 cells were treated with 45 nM AUY922 ± 2 µM VE821 for 24 h and a quantitative whole proteome analysis was done by mass spectrometry from 3 individual experiments. Volcano plots show highly altered proteins (cutoff: q<0.05; log2-fold change >0.5) after AUY922 (A), VE821 (B) and their combination (AUY922+VE821) (C). (D) A principal component analysis (PCA) was done on the proteome data set using R studio. The three replicates of each treatment are shown together with their median value (biggest symbol): DMSO (control) in blue, AUY922 in pink, VE821 in green and AUY922+VE821 in magenta. The circles around each group were inserted by hand after PCA for better visual separation.
