# Supplementary material for: Cooperative treatment effectiveness of ATR and HSP90 inhibition in Ewing’s sarcoma cells
Source: Cell Biosci. 2021 Mar 20;11:57. doi: 10.1186/s13578-021-00571-y (PMC7981928; doi:10.1186/s13578-021-00571-y)
Supplement: Supplementary file 9 — Additional file 9: Figure S9. Impaired PI3K/AKT/mTOR signaling after AUY-VE. WE-68 (A) and otherwise isogenic p53 wild-type (wt) and p53 null (p53-/-) HCT116 (B) cells were treated with 45 nM AUY922 ± 2 µM VE821. Analysis of indicated proteins was done by Western blot after 24 h. Vinculin was used to control protein loading. Immunoblots are representative for at least two independent experiments. [file 13578_2021_571_MOESM9_ESM.pptx]

## Slide 1
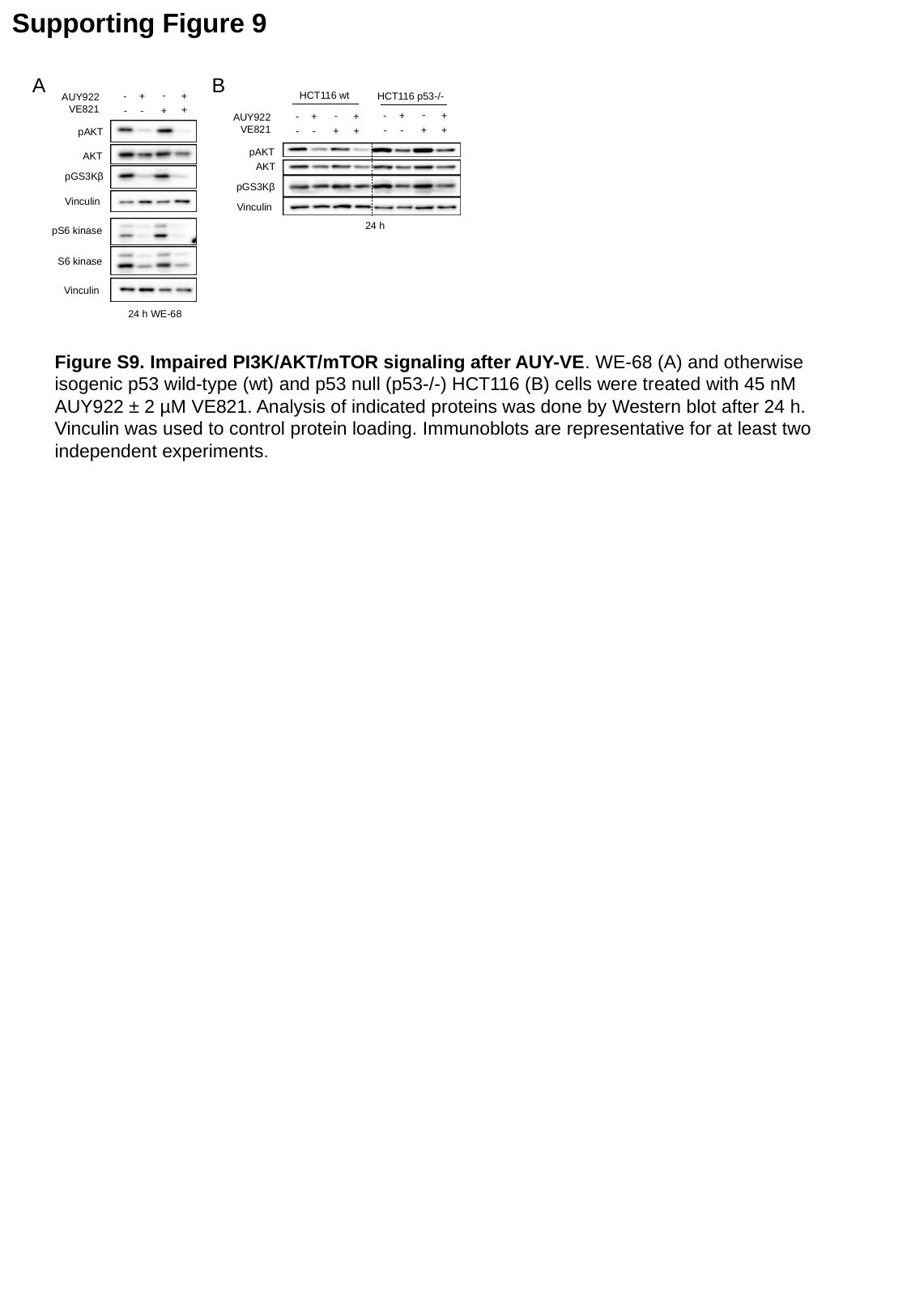

Supporting Figure 9
B
A
-
+
-
+
AUY922
VE821
+
-
-
+
pAKT
AKT
pGS3Kβ
Vinculin
pS6 kinase
S6 kinase
Vinculin
24 h WE-68
HCT116 wt
HCT116 p53-/-
-
-
+
-
+
+
-
+
AUY922
VE821
+
-
-
+
+
-
-
+
pAKT
AKT
pGS3Kβ
Vinculin
24 h
Figure S9. Impaired PI3K/AKT/mTOR signaling after AUY-VE. WE-68 (A) and otherwise isogenic p53 wild-type (wt) and p53 null (p53-/-) HCT116 (B) cells were treated with 45 nM AUY922 ± 2 µM VE821. Analysis of indicated proteins was done by Western blot after 24 h. Vinculin was used to control protein loading. Immunoblots are representative for at least two independent experiments.
