# Supplementary material for: Cooperative treatment effectiveness of ATR and HSP90 inhibition in Ewing’s sarcoma cells
Source: Cell Biosci. 2021 Mar 20;11:57. doi: 10.1186/s13578-021-00571-y (PMC7981928; doi:10.1186/s13578-021-00571-y)
Supplement: Supplementary file 10 — Additional file 10: Figure S10. Ingenuity pathway analysis of the proteome data set. A673 cells were treated with 45 nM AUY922 ± 2 µM VE821 for 24 h, and a quantitative whole proteome analysis was done by mass spectrometry from three individual experiments. The proteome data set was further processed by ingenuity pathway analysis (IPA; cutoff: q < 0.05) for toxicities after AUY922 (A) or VE821 (B) treatments. The common set of 208 overlapping differentially expressed proteins (DEPs) between AUY922-VE821 combinations (AUY-VE) and single treatments was processed by IPA for diseases and biofunctions (C) and toxicities (D). The unique set of 630 DEPs after AUY-VE treatments was processed by IPA for diseases and biofunctions (E) and toxicities (F). [file 13578_2021_571_MOESM10_ESM.pptx]

## Slide 1
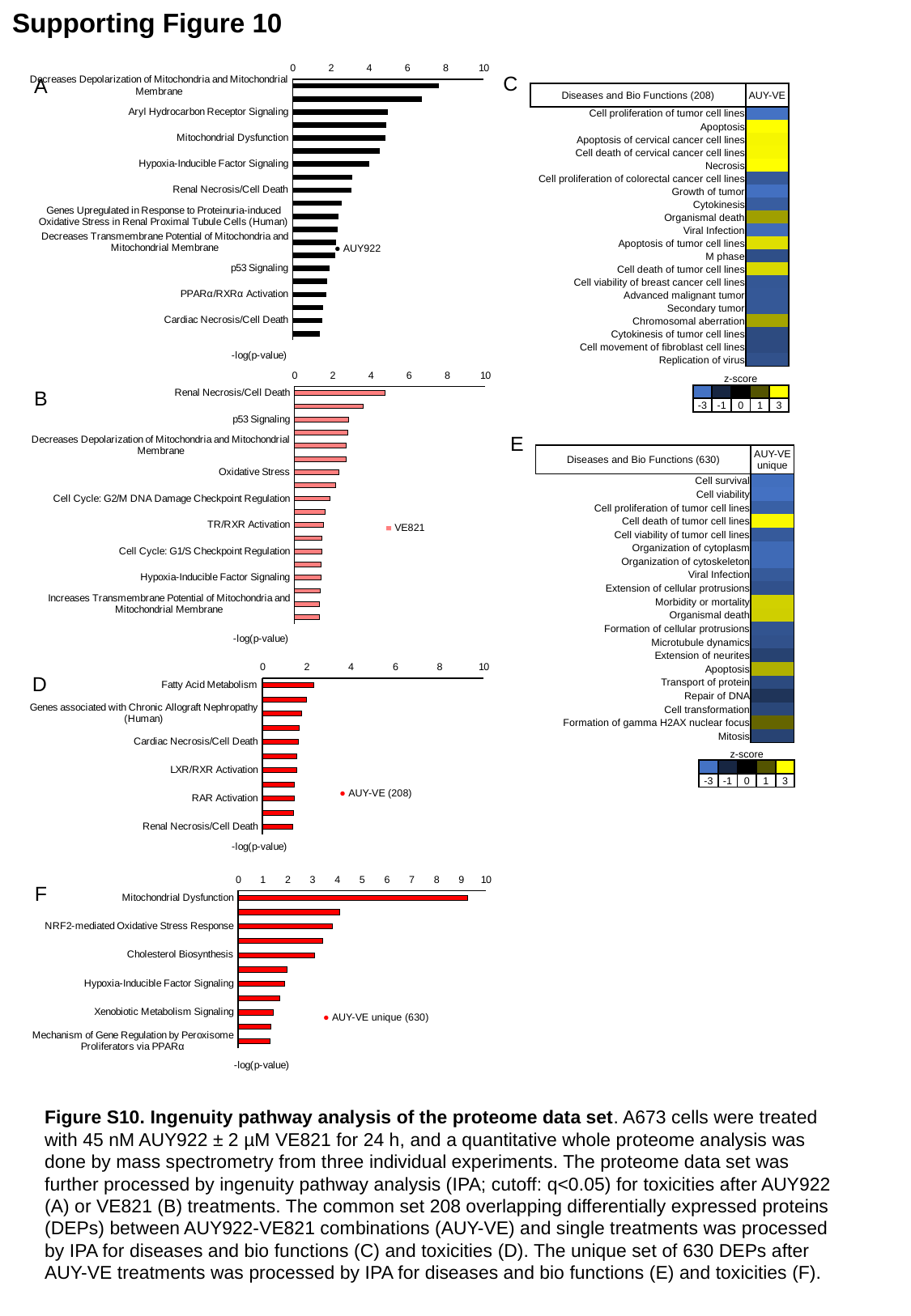

Supporting Figure 10
### Chart
| Category | |
|---|---|
| Decreases Depolarization of Mitochondria and Mitochondrial Membrane | 7.62 |
| NRF2-mediated Oxidative Stress Response | 6.75 |
| Aryl Hydrocarbon Receptor Signaling | 4.97 |
| Cell Cycle: G2/M DNA Damage Checkpoint Regulation | 4.86 |
| Mitochondrial Dysfunction | 4.85 |
| Fatty Acid Metabolism | 4.55 |
| Hypoxia-Inducible Factor Signaling | 3.98 |
| Decreases Permeability Transition of Mitochondria and Mitochondrial Membrane | 3.09 |
| Renal Necrosis/Cell Death | 3.06 |
| Increases Transmembrane Potential of Mitochondria and Mitochondrial Membrane | 2.55 |
| Genes Upregulated in Response to Proteinuria-induced Oxidative Stress in Renal Proximal Tubule Cells (Human) | 2.39 |
| Cholesterol Biosynthesis | 2.35 |
| Decreases Transmembrane Potential of Mitochondria and Mitochondrial Membrane | 2.25 |
| Renal Safety Biomarker Panel (PSTC) | 2.19 |
| p53 Signaling | 1.91 |
| Liver Necrosis/Cell Death | 1.79 |
| PPARα/RXRα Activation | 1.74 |
| Acute Renal Failure Panel (Rat) | 1.59 |
| Cardiac Necrosis/Cell Death | 1.55 |
| Primary Glomerulonephritis Biomarker Panel (Human) | 1.39 |C
A
| Diseases and Bio Functions (208) | AUY-VE |
| --- | --- |
| Cell proliferation of tumor cell lines | |
| Apoptosis | |
| Apoptosis of cervical cancer cell lines | |
| Cell death of cervical cancer cell lines | |
| Necrosis | |
| Cell proliferation of colorectal cancer cell lines | |
| Growth of tumor | |
| Cytokinesis | |
| Organismal death | |
| Viral Infection | |
| Apoptosis of tumor cell lines | |
| M phase | |
| Cell death of tumor cell lines | |
| Cell viability of breast cancer cell lines | |
| Advanced malignant tumor | |
| Secondary tumor | |
| Chromosomal aberration | |
| Cytokinesis of tumor cell lines | |
| Cell movement of fibroblast cell lines | |
| Replication of virus | |
 ● AUY922
### Chart
| Category | |
|---|---|
| Renal Necrosis/Cell Death | 4.76 |
| Mitochondrial Dysfunction | 3.62 |
| p53 Signaling | 2.82 |
| Anti-Apoptosis | 2.79 |
| Decreases Depolarization of Mitochondria and Mitochondrial Membrane | 2.73 |
| PXR/RXR Activation | 2.72 |
| Oxidative Stress | 2.35 |
| RAR Activation | 2.15 |
| Cell Cycle: G2/M DNA Damage Checkpoint Regulation | 1.87 |
| Decreases Transmembrane Potential of Mitochondria and Mitochondrial Membrane | 1.6 |
| TR/RXR Activation | 1.54 |
| Cardiac Necrosis/Cell Death | 1.46 |
| Cell Cycle: G1/S Checkpoint Regulation | 1.45 |
| Fatty Acid Metabolism | 1.4 |
| Hypoxia-Inducible Factor Signaling | 1.4 |
| Cardiac Hypertrophy | 1.35 |
| Increases Transmembrane Potential of Mitochondria and Mitochondrial Membrane | 1.33 |
| Increases Liver Hyperplasia/Hyperproliferation | 1.32 || z-score | | | | |
| --- | --- | --- | --- | --- |
| | | | | |
| -3 | -1 | 0 | 1 | 3 |
B
E
| Diseases and Bio Functions (630) | AUY-VE unique |
| --- | --- |
| Cell survival | |
| Cell viability | |
| Cell proliferation of tumor cell lines | |
| Cell death of tumor cell lines | |
| Cell viability of tumor cell lines | |
| Organization of cytoplasm | |
| Organization of cytoskeleton | |
| Viral Infection | |
| Extension of cellular protrusions | |
| Morbidity or mortality | |
| Organismal death | |
| Formation of cellular protrusions | |
| Microtubule dynamics | |
| Extension of neurites | |
| Apoptosis | |
| Transport of protein | |
| Repair of DNA | |
| Cell transformation | |
| Formation of gamma H2AX nuclear focus | |
| Mitosis | |
■ VE821
### Chart
| Category | |
|---|---|
| Fatty Acid Metabolism | 2.32 |
| Cholesterol Biosynthesis | 2.01 |
| Genes associated with Chronic Allograft Nephropathy (Human) | 1.78 |
| Mitochondrial Dysfunction | 1.65 |
| Cardiac Necrosis/Cell Death | 1.64 |
| Increases Liver Hyperplasia/Hyperproliferation | 1.56 |
| LXR/RXR Activation | 1.54 |
| Anti-Apoptosis | 1.44 |
| RAR Activation | 1.44 |
| Decreases Depolarization of Mitochondria and Mitochondrial Membrane | 1.42 |
| Renal Necrosis/Cell Death | 1.37 |D
| z-score | | | | |
| --- | --- | --- | --- | --- |
| | | | | |
| -3 | -1 | 0 | 1 | 3 |
 ● AUY-VE (208)
### Chart
| Category | |
|---|---|
| Mitochondrial Dysfunction | 9.27 |
| Renal Necrosis/Cell Death | 4.1 |
| NRF2-mediated Oxidative Stress Response | 3.82 |
| Cardiac Necrosis/Cell Death | 3.42 |
| Cholesterol Biosynthesis | 3.08 |
| Renal Glomerulus Panel (Human) | 1.96 |
| Hypoxia-Inducible Factor Signaling | 1.87 |
| Oxidative Stress | 1.67 |
| Xenobiotic Metabolism Signaling | 1.43 |
| RAR Activation | 1.33 |
| Mechanism of Gene Regulation by Peroxisome Proliferators via PPARα | 1.3 |F
 ● AUY-VE unique (630)
Figure S10. Ingenuity pathway analysis of the proteome data set. A673 cells were treated with 45 nM AUY922 ± 2 µM VE821 for 24 h, and a quantitative whole proteome analysis was done by mass spectrometry from three individual experiments. The proteome data set was further processed by ingenuity pathway analysis (IPA; cutoff: q<0.05) for toxicities after AUY922 (A) or VE821 (B) treatments. The common set 208 overlapping differentially expressed proteins (DEPs) between AUY922-VE821 combinations (AUY-VE) and single treatments was processed by IPA for diseases and bio functions (C) and toxicities (D). The unique set of 630 DEPs after AUY-VE treatments was processed by IPA for diseases and bio functions (E) and toxicities (F).
